# Guidance on selecting and evaluating AI auto-segmentation systems in clinical radiotherapy: insights from a six-vendor analysis

**DOI:** 10.1007/s13246-024-01513-x

**Published:** 2025-01-13

**Authors:** Branimir Rusanov, Martin A. Ebert, Mahsheed Sabet, Pejman Rowshanfarzad, Nathaniel Barry, Jake Kendrick, Zaid Alkhatib, Suki Gill, Joshua Dass, Nicholas Bucknell, Jeremy Croker, Colin Tang, Rohen White, Sean Bydder, Mandy Taylor, Luke Slama, Godfrey Mukwada

**Affiliations:** 1 School of Physics, Mathematics and Computing, The University of Western Australia, Crawley, WA Australia; 2 Department of Radiation Oncology, Sir Charles Gairdner Hospital, Nedlands, WA Australia; 3 Center for Advanced Technologies in Cancer Research, Perth, WA Australia; 4 Australian Centre for Quantitative Imaging, The University of Western Australia, Crawley, WA Australia; 5 School of Medicine and Public Health, University of Wisconsin, Madison, WI USA

**Keywords:** Selection criteria, Artificial intelligence, Automatic segmentation, Radiotherapy, Recommendations, Clinical evaluation

## Abstract

Artificial Intelligence (AI) based auto-segmentation has demonstrated numerous benefits to clinical radiotherapy workflows. However, the rapidly changing regulatory, research, and market environment presents challenges around selecting and evaluating the most suitable solution. To support the clinical adoption of AI auto-segmentation systems, Selection Criteria recommendations were developed to enable a holistic evaluation of vendors, considering not only raw performance but associated risks uniquely related to the clinical deployment of AI. In-house experience and key bodies of work on ethics, standards, and best practices for AI in Radiation Oncology were reviewed to inform selection criteria and evaluation strategies. A retrospective analysis using the criteria was performed across six vendors, including a quantitative assessment using five metrics (Dice, Hausdorff Distance, Average Surface Distance, Surface Dice, Added Path Length) across 20 head and neck, 20 thoracic, and 19 male pelvis patients for AI models as of March 2023. A total of 47 selection criteria were identified across seven categories. A retrospective analysis showed that overall no vendor performed exceedingly well, with systematically poor performance in Data Security & Responsibility, Vendor Support Tools, and Transparency & Ethics. In terms of raw performance, vendors varied widely from excellent to poor. As new regulations come into force and the scope of AI auto-segmentation systems adapt to clinical needs, continued interest in ensuring safe, fair, and transparent AI will persist. The selection and evaluation framework provided herein aims to promote user confidence by exploring the breadth of clinically relevant factors to support informed decision-making.

**Supplementary Information:**

The online version contains supplementary material available at 10.1007/s13246-024-01513-x.

## Introduction

Contouring (also referred to as “segmentation”) is a fundamental process in radiation oncology treatment planning; however, it is highly subjective and time consuming when performed manually. Variability between and within observers has been well documented [1], with errors in contouring leading to worse patient outcomes and greater organ-at-risk (OAR) toxicities [2, 3]. Manual contouring is a resource-intensive task, with times for head and neck (HN) contouring of six OARs taking up to 108 min [4]. Artificial intelligence (AI) based auto-segmentation has emerged as a promising solution to address these issues by offering reduced contouring time, improved contour consistency and accuracy, and decreased inter-observer variability (IOV) [5].

With the maturation of the AI based auto-segmentation market, a greater demand for clinical adoption in radiation therapy departments has emerged. However, multiple factors have limited the uptake of this new technology into clinical practice, including: (1) lack of a cohesive and holistic framework for product evaluation [6]; (2) a lack of domain expertise to conduct performance evaluation and ongoing quality assurance (QA) [6, 7]; (3) an absence of features for ensuring model safety [6–8].

Recently several works have emerged on AI ethics, standards, and best practices [8–21], however, firsthand clinical experiences evaluating and translating AI-based auto-segmentation systems into the clinical radiotherapy workflow are hitherto lacking. This paper aims to develop a framework for the selection of auto-segmentation systems in the evolving regulatory and market landscape. The selection criteria presented in Sect. 2 were retrospectively applied to six commercial systems in Sect. 3, which included a comprehensive quantitative evaluation to contribute to the existing literature on expected model performance. Using these recommendations, radiotherapy departments can be better positioned to confirm the safety, effectiveness, suitability, and efficiency of auto-segmentation software prior to clinical deployment.

### Fundamental principles and concepts

As outlined in IAEA-TCS 83, the roles and sponsibilities of Medical Physicists, as part of a wider AI implementation team, include the preparation of specifications, literature review, identification of potential vendors, considerations into workflow compatibility, safety and associated risks, regulatory compliance, and technical suitability as part of the procurement process [9]. Therefore, to properly evaluate any AI-based medical software, the implementation team must first understand the unique nature and challenges associated with AI model development and clinical deployment. Considerations include:

#### Model type

AI based segmentation models are deep neural networks which utilize convolutional operations, typically in a U-Net style architecture to automatically learn spatial hierarchies of features [21]. Models are typically trained on 3D data (input: 3D medical image, output: segmentation mask and/or DICOM-RT structure file) in a supervised manner. Supervision requires expert defined masks serving as ground truth or reference standard contours. A single model may be trained to predict several structures, or only a single structure per model. Peripheral models may be included in the framework to initially identify the anatomical region of the input scan prior to segmentation. Continuous learning models, which should be run in parallel to clinical models [16] and are currently not approved for clinical use [20], are able to learn from new institutional data and adapt on the fly. Similarly, some static models can be fine-tuned to local data characteristics prior to deployment.

#### Importance of data

The quality, quantity, diversity, and representativeness of data used to train and evaluate deep learning models is of central importance for ensuring robust clinical performance. Data used for model training must be sufficiently *diverse* with respect to population characteristics (age, weight, gender etc.) and scanner settings (manufacturer, acquisition protocols etc.). Data must also be sufficiently *representative* of the intended use case, for example, the specific case of paediatric pelvic organ-at-risk (OAR) segmentation must have sufficient examples in the training set [16, 19, 20]. Data association between training and evaluation sets must be completely independent, and not used for fine-tuning of the model [21]. Following the garbage-in, garbage-out aphorism, the *quality* of training data determines model competency. This includes any preprocessing and data cleaning for images, and reference segmentation guidelines followed (including accountability for intra and inter-observer variability) in ground truth segmentation formation [8, 22]. Inconsistency in any of these factors can result in performance degradation with respect to its intended use. Evaluation of model performance, whether conducted internally or externally to the organization, must adhere to the same standards of data quality, diversity, and representativeness as those required for its intended use.

#### How are models evaluated?

Segmentation models are evaluated based on the desired clinical endpoint and available resources. Quantitative metrics for contour evaluation can be broadly grouped into volume-based, distance-based, and surface-based approaches [5, 23]. Taha et al*.* undertook a comprehensive analysis of 20 metrics. They concluded that distance-based metrics such as Hausdorff Distance (HD) were better suited for both boundary agreement and general alignment assessments over volume-based metrics, and that they should always accompany volume-based metrics [23, 24]. Recent studies have shown that surface-based metrics such as Added Path Length [25, 26] and Surface Dice [26, 27] better correlate with clinically-relevant endpoints such as contour time-saving and contour acceptability [5, 28]. Contrarily, volume-based metrics have shown weak associations with clinical utility [5, 25, 26, 28–30], yet the Dice coefficient is the most widely reported metric in the literature [31]. An overview of metrics is summarized in Table 1.

**Table 1:** Descriptive summary of each quantitative metric used in the present study, with original definitions outlined in the cited publications

Metric	Description	Advantages	Disadvantages
DSC [67]	Measures the volumetric overlap between two structures with 1 signifying perfect overlap and 0 signifying no overlap	Easy implementation and interpretation Ubiquitous use in literature Normalized, allowing relative comparison between structures	Bias toward the volume of a structure Insensitive to boundary complexity Not well correlated with manual contour-correction time
HD95 [68]	HD95 is computed by finding the 95th percentile of all minimum distances between two contours. Alternatively, ASD takes the minimum distances of all points and computes the average	Distance metrics represent a real value comparable to clinically relevant data such as margins Boundary accuracy is better characterized than DSC	Insensitive to error type (small overall misalignment versus singular large error may result in same metric value, but have vastly different correction time burden) Not well correlated with manual contour-correction time
ASD [23]			
S-DSC [27]	Measures the perimeter overlap between contours within a set threshold (addition of a margin). S-DSC of 1 indicates perfect perimeter overlap, while 0 corresponds to no overlap	Tolerance parameter crudely accounts for IOV, and small clinically insignificant errors Is normalized, allowing relative comparisons between structures Well correlated with manual contour-correction time	Not widely used Tolerance threshold should vary with spatial position and organ to truly account for IOV
APL [25]	Considers the manual adjustment required, in voxels, to shrink or expand the auto-contour to match the ground truth within a set tolerance (addition of a margin)	Tolerance parameter crudely accounts for IOV, and small clinically insignificant errors Can provide absolute or surrogate estimate for contour correction time Well correlated with manual contour-correction time	Not widely used Tolerance threshold should vary with spatial position and organ to truly account for IOV Difficult implementation

Clinical assessments include dosimetric comparisons and efficiency/time-saving quantification. Dosimetric analyses have shown large heterogeneity in study design where manual contours are compared to either corrected or uncorrected auto-contours [31]; and the same treatment plans are either retained for both contour sets, or new optimizations are conducted based on the different contours [5, 31]. In each case, the dose-volume histogram is compared for each plan using metrics relevant to the assessed structure. Efficiency assessments measure the time benefit gained by using auto-contouring over manual contouring to produce a clinically acceptable plan. Time benefit is practically computed by considering the user time required to import auto-contoured structures into the treatment planning system (TPS) and edit them, minus the time required to manually contour the required structures from scratch [28].

Qualitative measures for clinical acceptability are typically performed using either the Likert scale (ranking system based on predetermined criteria) or Turing test (blind judgement whether contour is manually or automatically generated) [5, 28, 31, 32]. No consensus on quality score reporting exists, with literature quality assessment scales ranging from 2 to 11, along with heterogeneity in scoring instructions which vary between “clinical acceptability”, “contour source”, “estimated utility”, and “satisfaction rating” [31]. Although qualitative measures attempt to mimic real clinical judgement, operator subjectivity is still an issue, with inconsistencies in scoring demonstrated in several studies [5, 28, 31].

#### Bias, fairness and generalizability

Bias refers to the systematic underperformance of AI systems based on underlying differences in training, evaluation and clinical datasets, as well as operator and developer prejudice. If not identified and minimized, biases can exacerbate inequality in healthcare for under-represented groups [20], and infringe upon the fundamental human rights concerning equitable access to AI in medicine [9]. Biased AI is by definition unfair, and does not generalize well, therefore, identifying the most accurate model for a task is akin to selecting the least biased model.

Although up to 29 sources of bias have been identified in the AI development to deployment pipeline [33], five crucial biases in segmentation tasks can be summarized as follows [8, 20]:

- Population bias—patient characteristics between training and use-case differ (e.g. age or gender).
- Technical bias—differences in imaging characteristics or preprocessing between training and use-case (e.g. scanner vendor or image spacing).
- Annotation bias—differences in segmentation reference guidelines, experience of annotators, number of annotators and aggregation method, and extent of inter-observer variability between training and evaluation datasets.
- Clinical prevalence bias—training and use-case data have different proportions of patients with unique clinical factors (e.g. metal implants, rectal gel spacers etc.).
- Automation bias—clinical decision-making favours AI output contrary to other reliable data.

It is important to note that biases can emerge over time due to *drift* in the characteristics of the user data relative to the training data, a phenomenon called dataset drift [19].

#### Ethical and regulatory considerations

Ethics in AI is concerned with the preservation of values that align with broader ideals in medicine, which are summarily summarized in a recent RANZCR publication [13]. The concept of *transparency* in AI is important due to its “black box” nature and encompasses both *interpretability* and *explainability* which are important factors from both ethical and regulatory perspectives [20]:

- Transparency in AI refers to open dissemination of all aspects relevant to the system’s function (e.g. training data, model information, known limitations, intended use etc*.*)
- Interpretability is the ability to understand how the system functions in order to come to a decision (e.g. the inner workings of a specific deep learning model).
- Explainability refers to the system’s ability to provide an explanation for its decision (e.g. heat maps which correspond to information used by the model to make a prediction)

Regulations, although not uniform across nations nor exhaustive, are practical applications of these ethical principles. Both the European Union (EU) and United States (US) have developed regulatory frameworks for data and patient privacy protections, namely the General Data Protection Regulation (GDPR) and the Health Insurance Portability and Accountability Act (HIPAA), respectively [10, 17, 19].

When AI is treated as a medical device, it must meet quality standards defined under relevant jurisdictions. In the EU, compliance with the Medical Device Regulation (MDR) is required for CE clearance and the ability to market the product. The recent EU AI Act is the first comprehensive legal framework on AI [17], which entered into force on August 1, 2024, and uses a classification system to determine compliance requirements. Devices covered under MDR will need reassessment under the more stringent requirements of the AI Act, where vendors will be given a transitionary period to demonstrate compliance after the act becomes applicable on August 2, 2026 [34]. In the US, Food and Drug Administration (FDA) clearance is required to market AI for clinical use. The Australian Therapeutic Goods Administration (TGA) has recently implemented reforms to more closely align with US and EU regulations, where all three bodies now utilize a classification system to determine evidence requirements for compliance [35].

Recent industry standards for transparency in AI systems (IEEE P7001), data privacy (IEEE P7002), and bias control (IEEE P7003) have been defined [36], along with more comprehensive standardization through the AI development pipeline (ISO/IEC JTC 1/SC 42) [37]. Compliance with these standards can further promote trust in the ethical development of AI systems.

## Selection criteria

Recent publications on recommendations for best practices, ethical principles in the development and clinical deployment of AI, standards of practice for AI in radiotherapy, and in-house experience evaluating six commercial products were leveraged in formulating the selection criteria presented in Table 2 [8–21]. The specific requirements of the auto-segmentation system will vary for each institution; however, effort has been made to cover the breadth of clinically relevant factors that may influence the evaluation outcome.

**Table 2:** AI auto-contouring selection criteria guidance for clinical evaluation

	Criteria	Sub-criteria
1.1	Integration	Workflow disruption and operational efficiency
1.2		Developed with input from clinicians
1.3		Integration with existing systems & structure import (Filesystem, API, Web Interface)
1.4		Hardware requirements, scalability, and processing time
1.5		Flexibility of manual intervention
1.6		System updates (version control, rollback, continuous learning)
1.7		Vendor training and Support Services (Including Local Fine-tuning)
1.8		Template handling (Predefined, Custom, Multi-template Initiation)
1.9		Naming convention customizability and compliance
1.10		RT ROI identification code support (RapidPlan Compatibility)
2.1	Input Data & Use Cases	Total number of structures and clinical significance thereof
2.2		Ability to contour target volumes
2.3		Compatible imaging modalities (4D-CT, Average-CT, Low-dose CT, Dual-energy CT, Synthetic CT, CBCT, MRI and Sequences)
2.4		Compatible imaging vendors
2.5		Compatible acquisition settings (FOV, Image Spacing, Slice Thickness, Total Volume Size, MRI Field Strength, kV/mAs, Reconstruction Algorithm)
2.6		Artefact handling
2.7		Temporal consistency
2.8		Multi-modality contouring
2.9		Population suitability (Age, Size, Height, Gender, Ethnicity, Disease Burden)
2.10		Clinical factors (Contrast enhancement, abnormal setup, immobilization devices, bolus, brachytherapy applicators, abnormal anatomy)
3.1	Data Security & Responsibility	Local or cloud installation
3.2		Physical location of servers & compliance with local laws
3.3		Data encryption and de-identification
3.4		Compliance with standards & regulations
3.5		Cybersecurity best-practices
3.6		Data ownership
3.7		Learning on local data
3.8		Data minimization
4.1	Vendor Support Tools	Routine QA batch testing
4.2		Patient specific and automated QA
4.3		Output explainability function
4.4		Automated monitoring, logging, and error handling
4.5		Contour review requirements
4.6		Contour editing tools
4.7		Supplementary functionality
4.8		Research mode
5.1	Billing Structure	Pricing model (per Linac, per Scan, One-time Fee, Ongoing Subscription)
5.2		Additional costs (Cloud Hosting, Proprietary Hardware, Local Server Maintenance, Local Training, Updates, Support)
6.1	Transparency and Ethics	Model facts sheet (Intended Use Case, Limitations, Biases, Failure-modes, Evidence Gaps)
6.2		Information on model interpretability
6.3		Training and testing data characterization
6.4		Expected performance and tolerances
6.5		Quality control in ground truth formation
6.6		Regulatory approval
6.7		Risk management considerations (Bias, Dataset Drift, Product Lifecycle, Post-market Surveillance)
7.1	Performance Evaluation	Demo trial available for end-to-end testing
7.2		Evaluation (Quantitative, Qualitative, Time-saving, Dosimetric)

### Integration

Effective integration of the auto-contouring system should be seamless, resulting in minimal disruption of the existing workflow. Where there are changes, the extent of additional operations such as manual interaction, clicks, and window switching should be minimized to ensure operational efficiency. Ideally, the system should have been developed with input from multidisciplinary clinicians to achieve these goals. The system’s integration with existing software such as treatment planning systems (TPS) should be determined, with some vendors offering structure import and interaction through TPS plug-ins via API functionality, while others offer web interface or filesystem import options.

Hardware requirements such as network speeds and associated bottlenecks, storage, and GPU/CPU/RAM must be considered whether opting for cloud or local installations. For local installations, vendors may only permit installation on proprietary hardware, or allow for the use of existing servers. For cloud solutions, network bandwidth and connection reliability become critical. In addition, the local and/or cloud configuration must be scalable for future workload demands. Given the available hardware, an estimate of the typical processing time to output a segmentation must be determined.

The capacity to manually intervene in the auto-contouring workflow to troubleshoot issues must be considered when opting for cloud solutions compared to local installations, with the latter generally having greater flexibility. Similarly, system maintenance and update handling must be well understood. Version control, parallel version installations, and version rollback capacity must be considered, since re-evaluation of the AI system is recommended after each update [12]. These considerations are especially important for models that can continuously learn.

Any vendor training and support services must be understood, outlining typical response times and support availability. For models that can be fine-tuned to local population characteristics and imaging protocols, explicit instructions and training should be provided to ensure optimal data preparation and model retraining.

The extent of template customization for both specific structures and structure names allows users to fine-tune their auto-contouring outputs as desired at the cost of increased complexity. Some centers may find it useful to reduce the number of structures generated to prevent network slow-downs, improve model output speeds, and reduce TPS structure clutter. The ability to initiate multiple templates for a single scan or re-run the same patient using a different template can reduce costs if paying on a per-scan basis. For clinics operating Eclipse TPS, support for structure codes (RT ROI Identification Code) is useful when using RapidPlan. Centers may also benefit from structure naming conventions to be compliant with published standards such as in AAPM TG-263 [38].

### Input data & use cases

It is important to define the exact requirement the end user desires from the software. Greater accuracy and efficiency of segmentation output is typically assumed for capable AI systems, however, clinics may consider additional workflows such as adaptive radiotherapy, MR-only radiotherapy, and response assessment—each of which carry unique input data requirements. Furthermore, to ensure bias-free and generalizable outputs, users must consider a range of populations characteristics, clinical characteristics, and technical characteristics that may be relevant for their specific use case. As a first step, users must verify whether any input data restrictions have been put in place as safety measures, for example, age-ranges.

Users must consider the number of supported structures (including sub-structures), but more importantly, the significance of certain structures over others. For example, the ability to auto-contour nodal regions is advantageous for clinical target volume (CTV) definition and may take higher priority over the ability to contour more organs-at-risk (OAR).

The range of imaging modalities of interest may span CT (4D-CT phases, average-CT, low-dose CT, dual-energy CT), Synthetic CT, CBCT, and MRI including the variety of relevant sequences. Furthermore, institutions may utilize one or more imaging vendors and image reconstruction algorithms. The resulting images may be further characterized based on acquisition settings (field of view, voxel resolution, slice thickness variation, total volume size, MRI field strength, and kV/mAs). The prevalence of artefacts may also affect model performance, for example the presence of metal artefacts and the use of metal artefact reduction algorithms. The ability to integrate multiple imaging modalities simultaneously (i.e. PET/CT) for more accurate segmentations may also be considered. Additionally, the temporal consistency of the model, for example across CBCT or MR acquisitions must be verified if adaptive applications are planned.

In terms of population variability, the user must consider software compatibility for age ranges, patient size (very small and very large), patient height, gender, and ethnicity. Furthermore, if segmentation of disease sites is required, then the extent of disease burden or tumor staging may be important.

Clinical factors that affect individual imaging variability may influence model performance. These include the use of contrast enhancement, abnormal setup (i.e. excessive patient tilt, extreme off-center positioning, prone setup, feet-first setup), the use of different immobilization devices with varying densities, the inclusion of bolus or brachytherapy applicators on the patient’s skin, and any abnormal anatomy (i.e. rectal spacer, cists, post-surgery patients).

### Data security and responsibility

Assurance that sensitive patient data is protected, and that patient confidentiality is maintained is a challenging task in the rapidly changing (and differing by jurisdiction) regulatory and cyber-threat environment. Foremost, the type of system installation determines the extent of security considerations: cloud-based solutions require a greater burden of demonstrable security. The physical location of servers is important as data sovereignty laws may require that data not leave the jurisdiction. Furthermore, the location of the cloud servers must comply with local data protection laws of the clinic’s location. Any data travelling to and from the cloud should be encrypted and de-identified. Local organizational and IT preferences for cloud or local installations should be consulted before proceeding.

While it may not be mandatory in the clinic’s region, adherence to data protection regulations such as GDPR in the EU and HIPAA in the US alongside local legislative requirements provides additional confidence in the vendors commitment to good data security practices. Additional certifications such as IEEE P7002 provide greater assurance of robust security measures [36]. Cybersecurity is a growing concern with devastating consequences such as data manipulation, data theft, and loss of institutional trust if not guarded against. Vendors must demonstrate commitment to best practices in cybersecurity, especially if cloud implementations are pursued. Compliance with standards such as ISO/IEC 27001 (Information Security Management Systems), ISO/IEC 27002 (Information Security Controls), and ISO/IEC 27018 (Protection of Personally Identifiable Information), demonstrate adherence to good practices for data security in cloud deployments [39].

Clarity on data ownership is important, especially for AI systems that use data for any kind of model training. Contractual agreements between clinic and vendor must be in place for the use of patient data which satisfy local and national regulations. Additionally, informed patient consent on the use of their data may be a legal necessity in the user’s jurisdiction. This includes both the use of AI on their data, but also the training of AI models. Provision for data minimization strategies should be available, allowing the user to fine-tune the amount of identifiable patient information that is uploaded to the cloud.

### Vendor support tools

Vendor support tools and supplementary functionality span a variety of features which enhance the user experience beyond the base auto-segmentation capabilities of the software. Quantitative evaluation of AI model accuracy is required for intercomparison, baselining, and *routine quality assurance* (QA) once integrated into clinical practice. A major impediment to clinical implementation of auto-segmentation models for busy departments or departments lacking coding expertise is the ability to evaluate segmentation performance quickly and accurately. Furthermore, it has been shown that in-house implementations of quantitative metrics typically contain bugs, with deviations of up to 200% between institutions [40]. Therefore, integrated tools for batch testing and performance logging can offer a standardized approach to expedite evaluation. In addition, the vendor may provide their own set of QA reference data and procedures prior to acceptance to verify proper software function.

Tools for automated *patient-specific QA* aim to increase the efficiency of contour review and reduce the onset of automation bias by directing user attention to poor outputs. For example, an input-data suitability checker can assess how well the input data compares to the training cohort characteristics. Out-of-distribution data can either be flagged or prevented from being used on the model. Uncertainty estimation methods can be used to spatially visualize and locate regions of high model uncertainty and direct user attention to them [41–43]. Alternatively, a clinical and verification model can be deployed in parallel on the same data, with large structure-wise or slice-wise differences in metrics flagged for user review [44, 45]. Other tools, such as saliency maps or confidence scores can be used to improve model explainability.

Beyond quantification of model performance, methods for monitoring, logging, and error handling/reporting are desirable. Automated logging of both routine and patient-specific QA results enables tracking of model performance trends over time, potentially highlighting the need for re-commissioning or troubleshooting due to dataset drift. Automated logging of throughput details (patient, version, time etc*.*) and error handling/reporting is essential for effective troubleshooting and documentation in the event of incidents, and may also be required to meet regulatory or auditing requirements.

Depending on the vendor, manual contour review may be required for each structure on a slice-by-slice basis, only for a single slice, the entire 3D render, or may not be required at all. Additionally, the ability to edit the generated segmentations and perform operations using vendor provided tools may be advantageous if the tools save time over traditional editing approaches.

Supplementary functionality, for example, may extend to adaptive radiotherapy (ART) applications or response assessment using criteria such as RECIST [46]. For ART, users must consider additional functionality such as rigid and deformable image registration, dose summation capabilities, synthetic CT generation, and rapid treatment planning with minimal workflow restrictions.

For research-focused clinics, a “research mode” allowing users to easily filter previously processed patients by certain criteria, anonymize contour data, and analyze or export the data in bulk may be regarded highly. The research environment may also allow experimentation with new features or versions while not impacting the clinical environment.

### Billing structure

The billing structure may be per linear accelerator (linac), per scan, a one-time fee, or an ongoing subscription where the most economical choice is unique for each institution. Users must consider additional fees associated with hosting data and models on cloud servers, setup and maintenance of local servers, model (re)training, updates, and service and support fees. In addition, in the event the user terminates their subscription, what grace periods exist, if any? In determining these factors, the user must be aware of their total patient throughput, including a buffer for re-scans and re-contouring due to user errors.

### Transparency and ethics

Deep learning algorithms intrinsically do not allow users to understand how decisions are made. Their capabilities are borne out of the underlying model architecture and data used to create them. Satisfying the core medical ethics principles—autonomy, nonmaleficence, beneficence, and justice [47]—which were recently adapted for medical AI through eight specific guidelines [13], demands that users have a deeper understanding of the strategies employed in model development.

The concept of a “model facts sheet” has been proposed to summarize algorithm information, purpose, and design principles in a standardized format [48]. These fact sheets allow users to readily understand the specific use-cases and patient populations for which the model was developed; and outline the conditions under which the model is not applicable or has known limitations, biases, failure-modes, or gaps (in both training and clinical evaluation datasets) [49]. Obtaining further technical information such as an overview of the underlying model architecture and its functionality, data pre- or post-processing techniques, use of synthetic data, and addressing concerns over model overfitting can enhance model interpretability and user ability to diagnose failure cases.

In no uncertain terms the user should be able to compare their specific use-case data (as outlined in Sect. “Input data & use cases”) to the data used for model training. This spans the specific population characteristics, technical characteristics, and clinical characteristics of the user institution data, paying close attention to the total number of datapoints for each criterion but also the relative distribution of those datapoints in the corresponding model training set. The importance of this comparison cannot be overstated as the performance of an AI, and by extension notions of fairness and equitability, are determined by the similarity of training and use-case data [20]. Tangentially important is the testing dataset on which the model evaluation has been performed. Whether internal or external, the testing set must not contain any data associated with the training set. The characteristics of the testing set must be equally well defined, ideally following best-practice reporting guidelines such as CLAIM [50], CONSORT-AI [51], DECIDE-AI [52], or SPIRIT-AI [53], allowing transparent determination of model performance. These internal and external evaluations help users understand the model's expected performance for their specific population, technical, and clinical characteristics. Ideally, these performance levels should be accompanied by tolerance levels for acceptable performance on the user’s own data.

Defining the ground truth or reference standard data is just as crucial as ensuring the diversity and representativeness of the training data. Segmentations are prone to bias based on the subjective experience of the observer. Factors owing to experience, fatigue, and the nature of the manual segmentation process itself result in notable contour variability between clinicians (inter-observer variability) and at different time points (intra-observer variability). Hence, the user should enquire about the total number of unique labelers, the number of labelers per patient, the experience of those labelers, the recognized reference guidelines used [22], and any quality control or data screening methods employed.

Clinical use of AI systems requires regulatory approval in the relevant jurisdiction. As previously highlighted, the FDA-approved version of the software should be compared to the user’s version as vendors may purposefully limit the capabilities of the US-approved version to ensure regulatory compliance [21]. Additionally, users may enquire about regulatory compliance documents, which may contain, for example, evidence of bias mitigation, strategies to limit dataset drift, and product lifecycle and post-market surveillance plans [35].

### Performance evaluation

Assessment of model performance on local data is a crucial step in the selection process for several reasons: local laws and rules may require proof of evaluation, product features and performance claims can be substantiated locally, biases and limitations can be identified, expectations and trust in the tool can be set, and workflow impacts can be quantified. For the evaluation, trial access to the software should be provided for comprehensive end-to-end testing. Alternatively, the vendor may ask for institutional data and return the automatically segmented structures without granting user access to the system itself. As a general principle, data characteristics for evaluation should be reflective of the practice’s typical distribution, and the amount of data used should be as large as reasonably achievable. A summary of auto-segmentation evaluation approaches is presented in Table 3, where green, yellow, and red represent the highest, moderate, and lowest level of evidence, respectively.

**Table 3:**
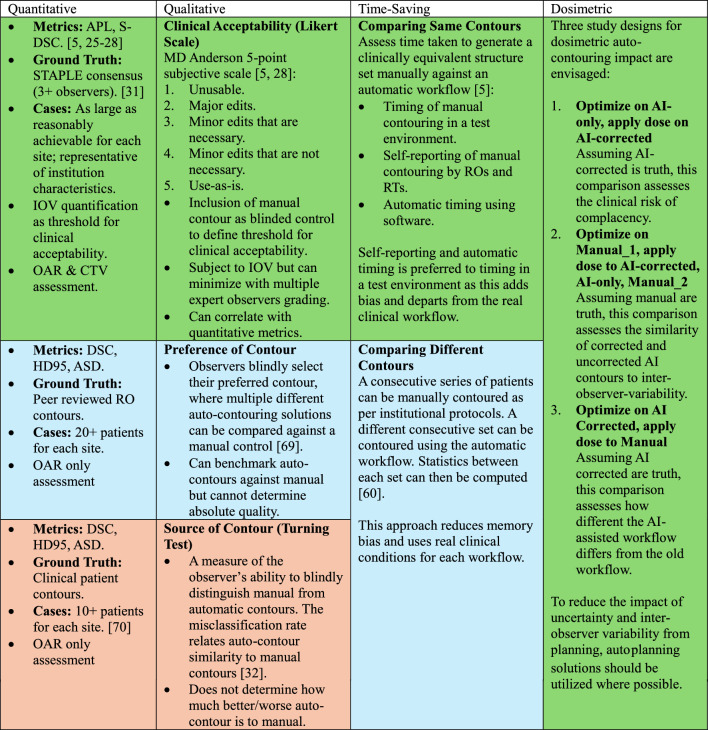
Recommendations for performance assessment of AI Auto-contouring Solutions

Colour coding suggests relative level of scientific evidence within category: green = greatest relevancy; yellow = moderate relevancy; red = least relevancy

In instances where limited clinical resources prevent the comprehensive evaluation of AI models on local data, several options may be considered: collaboration with institutions with similar equipment and treating similar patient demographics that are able to lend data for testing; or at minimum, comparing any internal or external model testing data to the user institution data, with particular attention paid to similarity of technical, clinical and population characteristics [20]. One should avoid using open-access data for model evaluation as it is possible some models were trained using the same data, thereby introducing bias into the evaluation.

#### Quantitative analysis

Quantitative analyses have the advantage of being readily performed on large amounts of data with reduced burden on clinical resources, however, they may not fully capture or reflect the clinical utility of the auto-segmentation model. For example, DSC, HD, and ASD have shown limited correlation with clinical acceptability of contours [6, 30, 54] or correlation with dosimetric outcomes [30]. Additionally, no evidence has demonstrated that a universal threshold (i.e. DSC of 0.7 [55], 0.8–0.9 [56]) exists to differentiate between poor- and high-quality contours [6, 55]. The recent introduction of two surface-based metrics, APL and S-DSC, have shown a moderate to strong correlation with contour correction time saving of 0.87 [25] and 0.69 [26] for APL, and − 0.69 [25] and − 0.48 [26] for S-DSC. Optimal S-DSC thresholds have also been shown to reflect contour quality to above 91% accuracy [44]. Therefore, the use of S-DSC and APL is recommended for quantitative evaluation. Furthermore, manual ground truth contours should ideally be of the highest quality to minimize IOV related bias. To achieve this, consensus contours from a minimum of three experts defined using the STAPLE (Simultaneous Truth and Performance Level Estimation) algorithm which combines multiple annotations using a probabilistic model [22, 24, 57]—as per RTOG reference volume creation practices—is recommended [22]. Multiple observer contours can also be used to quantify IOV, which can function as a metric threshold for clinical acceptability of auto-generated contours [58]. Alternatively, although less robust, manual contours can be peer-reviewed to ensure quality-control. Lastly, clinical contours may be used where time and resource constraints limit high-quality data accrual.

#### Qualitative analysis

Since the Radiation Oncologist (RO) is ultimately responsible for contour accuracy, it is highly recommended to include a qualitative assessment alongside other evaluation methods. When multiple evaluation approaches align with qualitative findings, clinicians can maintain greater confidence in their conclusions. Qualitative assessments are typically formed using either a Likert scale for clinical acceptability, a Preference of Contour approach, or Source of Contour (Turning Test) approach, as summarized in Table 3. A large range of Likert scales exist; however, a 5-point scale has been recommended in two recent reviews [28, 31] as it was better able to discriminate contour quality than a 3-point scale [5]. As such, qualitative assessment using the MD Anderson definition is recommended for greater standardization [28]. The inclusion of manual contours in the blind pool can be a useful benchmark for acceptability, while recruiting multiple observers helps to reduce IOV in scoring. The Preference of Contour [32] and Source of Contour [40] approaches both suffer from the same issue in that absolute contour quality cannot be determined.

#### Time-saving assessment

Time saving assessments place emphasis on the performance of the human-AI team, rather than the AI in isolation [59]. In Table 3, the AI-augmented workflow is compared with the manual workflow in terms of absolute and relative time saving to generate initial contours (by AI or Radiation Therapists (RTs)) and subsequently correct them (ROs). Previous studies have assessed time saving by creating test environments, using automatic timing software, or self-reporting [5]. Experiments that deviate least from the clinical workflow will most truthfully capture the real time burden, hence, the use of automatic timing software is recommended, followed by self-reporting, and lastly by the use of a test environment. Alternatively, a series of patients could be consecutively contoured using both the standard and AI-augmented workflows. After a large enough sample size for both groups has accumulated, time statistics can be computed and compared [60].

#### Dosimetric analysis

Three dosimetric assessment experiments are envisaged in Table 3 based on a literature survey [60] which compare different combinations of contours for optimization and assessment (manual, AI-only, and AI-corrected contours). The first test assesses the risk of complacency by optimizing plans on AI-only contours and comparing dose-volume statistics produced by applying the plan to AI-corrected contours. The second test assesses a plan created on a single manual contour set, to the same plan applied to AI-only, AI-corrected, and a second manual structure set. AI-only and AI-corrected structures can then be compared to IOV. The third test compares the AI-augmented workflow to the manual workflow by planning on the former and applying the dose to the latter. Where applicable, the use of auto-planning systems is recommended to reduce uncertainty in plan generation.

## In-house case study

At our institution, six commercial vendors were considered, of which one did not wish to be named. The other vendors, as assessed on March 2023, were Limbus AI v1.6.0, Radformation AutoContour v2.2.8, MiM Contour Protégé AI v7.2.8, Mvision AI v1.2.3, and Therapanacea ART-Plan v1.11.4-rc1. Vendor associations were removed to comply with privacy requests. De-identified vendors are randomly assigned as A-F hereafter. All models were trained on vendor datasets and used “as provided”, with no local fine-tuning performed.

### Application of selection criteria

At the time of consideration, available literature on guidance and best practices for selecting and implementing AI based auto-segmentation systems was nascent. This served as a limitation on the initial product assessment as most selection criteria were informed through discussions within our multidisciplinary team. In the time since the original assessment, parallel developments stemming from a growth in the literature and our own experience using AI auto-segmentation software daily has provided valuable insights into critical selection factors that were originally unconsidered.

A re-assessment using the recommended criteria for the six products as provided on March 2023 is summarized in Table 4, with each criterion scored from 1 to 5, where 1 is poor and 5 is excellent. Criteria scoring is determined based on our institutional priorities and available knowledge at the time of assessment. A weighting factor is assigned to each criterion to reflect the relative importance assigned by our clinic.

**Table 4:** Retrospective scoring of six vendors across the seven identified selection criteria

Criteria	Weight (%)	Vendor A	Vendor B	Vendor C	Vendor D	Vendor E	Vendor F
Integration	15	4.50	3.50	3.00	1.50	1.50	3.50
Input data & use cases	15	4.00	3.50	3.50	2.00	4.00	4.00
Data security & responsibility	20	3.50	1.00	3.50	1.00	1.00	2.00
Vendor support tools	5	1.00	2.50	1.00	1.00	1.00	3.00
Billing structure	20	4.00	4.50	3.50	4.00	1.00	3.00
Transparency & ethics	5	2.00	1.00	2.00	3.00	1.50	2.50
Performance evaluation	20	4.50	2.50	1.00	3.50	3.50	4.50
Weighted score /5		3.83	2.83	2.73	2.43	2.05	3.30

Weighting factor and individual scoring were determined based on institutional preferences. Scoring legend: 1 = poor; 5 = excellent

#### Integration

Our practice valued minimal disruption to the established Eclipse TPS-based workflow, and a large degree of template customization and local control for troubleshooting purposes. Vendor A provided the greatest degree of autonomy for the user to define custom contouring templates, custom file paths for image handling and transfer, and the only vendor at the time which gave the option to manually apply updates. Their solution was quick enough for our purposes and required no modification of existing hardware or software resources. It was also one of three vendors that supported RapidPlan structure code output. Other vendors partially satisfied these requirements but typically fell short with respect to seamless integration and the ability to customize templates to our preferences.

#### Input data & use cases

Our intention was to utilize auto-segmentation for enhanced efficiency primarily, and improved consistency as a secondary consideration. Hence, the ability to handle standard imaging parameters, including images with metal artefacts, typical immobilization devices, average CTs and varying slice thicknesses and FOV from a Toshiba Aquilion LB CT scanner (Canon Medical Systems, Otawara, Japan) was imperative. Support for a large number of OAR and nodal target structures was preferred. Vendors E and F supported the greatest number of structures at 149 and 150 respectively, while vendor A offered 100. Only vendor A and C made explicit comments regarding preferable CT vendors to use with their models, of which only vendor A made mention of Toshiba scanners. Likewise, only vendor A outlined potential underperformance for patients with uncommon posture, orientation, setup, or containing prosthetic devices. All vendors offered lymph node support other than vendor D for the head and neck; vendor D also contained the least number of structures at 51.

#### Data security & responsibility

Our hospital security services demand that strict cybersecurity and patient safety/privacy processes are in place, as such a high weighting factor was assigned to this category. Foremost, a local server implementation was highly desired, of which only vendors A, C, D, and F provided an option. However, only Vendor A could be run on existing local servers, which suited established security practices. All vendors were scant on information regarding compliance with data privacy standards and regulations, however, Vendor C did make mention of their commitment to HIPAA and GDPR regulations, adherence to certain IEC standards, and ability to choose data minimization options. Overall, information regarding the physical location of cloud servers, data collection and ownership statements, and data de-identification and encryption statements were mostly absent across all vendors.

#### Vendor support tools

At the time of assessment, the implementation team did not consider the possibility nor utility of vendor support tools, hence a low weight factor was assigned to this category. Most vendors did not offer much outside of auto-segmentation functionality and data logging. Vendor B integrated well with Eclipse and provided additional contour editing tools. They also required users to manually check all auto-contours prior to clinical use. Vendor F offered the greatest supplementary functionality, allowing auto-contouring integration with workflows for synthetic CT based MR-only treatment planning, and adaptive radiotherapy using deformable image registration. Unfortunately, no vendors offered tools to assist QA, evaluation, nor enhance model explainability.

#### Billing structure

The most cost-effective solution was one that minimized upfront costs, as well as additional costs relating to system setup and maintenance. Payment per linac was preferred as no limit would be placed on the number of segmentation outputs. There was a large variability in upfront pricing, however, most vendors were in a similar ballpark. The benefit of being able to utilize existing local hardware, as offered by Vendor A, without the need to upgrade or install proprietary servers meant appreciable cost savings compared to other solutions with respect to setup and maintenance and resulted in favorable scoring.

#### Transparency & ethics

Comprehensive investigations into transparency and ethics were not considered due to the paucity of guidance available at the time, hence, a low weighting factor was assigned to this category. Only vendors A, D, and F had regulatory approval in our country at the time of assessment, while Vendors B and E provided estimated dates for approval. Only vendor D provided quantitative data on internal model performance for a range of structures, as well as brief mention of the model architecture. Meanwhile, Vendor F made reference to several external literature evaluations. No vendors had model facts sheets prepared, information on training data characteristics, statements on risk management, or information on quality control for ground truth formation, meaning transparency and ethics was generally scored low for all options.

#### Performance evaluation

Ideal performance evaluation requires access to trial software to verify workflow considerations and to allow for a comprehensive evaluation of quantitative, qualitative, time-saving, and/or dosimetric performance. Only Vendors A, B, and D offered trial licenses, while the remaining vendors processed our institutional data through secure file transfer. We advise interested readers to consult the supplementary materials for specific information regarding our quantitative assessment which included 20 HN, 20 thoracic, and 19 male pelvis patients with institutionally relevant heterogeneity in image acquisition protocols and patient characteristics. Quantitative results are presented as box and whisker plots (Supplementary Figs. 2A–16A) for DSC, HD95, ASD, S-DSC (1mm margin), and APL (1mm margin) metrics, and are accompanied by a scoring heuristic which allowed for easy data interpretation (Supplementary Tables 3A–5A). Vendor performance across all sites, ranked from best-to-worst based on our scoring heuristic was A, F, E, D, B, C.

A blinded qualitative assessment was performed alongside the quantitative evaluation. Unfortunately, the study was not performed consistently across all observers, vendors, and patients due to clinical resource constraints. The findings are included in the supplementary materials as an adjacent study since expert opinion is a crucial component of the MDT selection process. Considering overall RO choice of vendor, there was agreement between qualitative and quantitative results for the HN (A/F) and thoracic sites (A), while no vendor was preferred for the pelvic region (see Supplementary Table 7A). RO scoring of individual OAR saw moderate to high Pearson correlation between qualitative and quantitative assessments as summarized in Supplementary Table 8A (HN: 0.741, thorax: 0.462, pelvis: 0.927). While differences were expected due to dissimilarity in sample sizes between qualitative and quantitative analyses, “best performing” vendors were accurately captured by both evaluation approaches. This general agreement between qualitative and quantitative results provided confidence in the assessment methodologies and allowed a clear decision to be made regarding our institution’s preferred vendor.

## Discussion

Selecting and evaluating AI based auto-segmentation systems in the rapidly evolving regulatory, research and market landscape is a challenging task. The current work provides guidance by exploring a nuanced set of criteria for the holistic assessment of AI auto-segmentation commercial solutions. Informed from clinical experience and available literature [8–21], the criteria aim to bring about the safest, fairest, least biased, and most efficient system under consideration.

Across the various criteria, we observed systematically lower scores for Data Security & Responsibility, Vendor Support Tools, and Transparency & Ethics, furthermore no single vendor scored exceedingly high overall. These observations suggest vendors are playing catch-up with both clinical and regulatory demands. The new EU AI Act regulations will likely become a standard for quality once enacted [61], and require manufacturers to better satisfy the hitherto lacking transparency and data security criteria. Meanwhile, clinical users of auto-segmentation systems—from both a utilitarian and safety perspective—would benefit from the greater integration of support tools. Investigations of automated QA methods are currently a hot research topic but are yet to be integrated into clinical practice [62]. Lower hanging fruit, however, such as a research mode or batch evaluation functionality may have more immediate impacts.

Raw model performance, in terms of quantitative metrics, varied widely from poor to excellent as summarized by the scoring heuristic in Supplementary Tables 3A–5A. Without greater transparency of the training datasets used to create these models, we can only speculate why performance varied so greatly. For these reasons, one cannot preclude the optimal model for their institution without a thorough performance evaluation, ideally following best practices as outlined in Table 3. Comparing our results to the wider literature, Supplementary Table 6A in the supplementary materials shows the median DSC ranges for 23 structures in relation to results from Doolan et al*.* [63]. Only three structures had non-overlapping ranges: prostate (0.722–0.826 vs. 0.854–0.905 [63]), heart (0.879–0.907 vs. 0.919–0.950 [63]), and femoral heads (0.943–0.966 vs. 0.883–0.913 [63]). Differences were attributed to varying superior-inferior extents and contouring practices. Despite minor discrepancies, overall vendor performance metrics aligned well, enabling prospective clinics to contrast their own assessments in the absence of expected model performance thresholds provided by the vendor.

### Clinical reality

Both positive and negative observations were noted after a year of using Vendor A. An internal survey was conducted 3-months into using the AI-augmented workflow, which found an average time reduction in OAR contouring of 51% across all sites. Meanwhile, negligible time saving was reported for target volume contouring. Informal discussions with RTs and ROs shared the view that the vast majority of OAR structures needed little to no corrections. Recent changes to TGA requirements meant that Vendor A was temporarily made unavailable for clinical use. Vendor B was provided as an alternative, with immediate clinical impact: a notable increase in the severity and frequency of manual corrections was noted, with one informal qualitative comparison rating Vendor A as 8/10 and Vendor B as 5/10 for segmentation accuracy. This underscores the importance of conducting comprehensive evaluation prior to selection.

Despite the positive feedback received at our institution, certain challenges and risks were identified. Workflow issues initially posed a risk in some cases, for instance, there was confusion whether automatically generated structures had been manually checked prior to plan approval. Another example is the risks arising from structure naming protocols. In one instance at our institution, the default AI structure name for lymph nodes was kept instead of renaming to the institutional standard (*i.e.* CTV48), resulting in a lower planned dose level to the nodes than the prescription. In addition, the quality of AI generated contours could pose risks to plan optimization if not corrected. Six potential fallibilities were identified: (1) incorrect naming of structures (higher risk for symmetric organs *e.g.* left eye named right eye); (2) structure continuity issues (*e.g.* slices missing); (3) empty structure sets; (4) partially contoured structures (*e.g.* kidneys half contoured); (5) holes in structures; (6) disconnected components; (7) catastrophically incorrect structures (*e.g.* stomach structure in the rectum).

As clinicians became accustomed to the new workflow, greater emphasis was placed on manual checking and removal of structures not used for optimization. As familiarity with the system grew, clinicians could anticipate for which sites and patients the software was more likely to underperform (for instance, pediatric patients, sites with missing tissue, sites with abnormal cist growths, and HN nodal volumes which almost always needed adjustments). Similar experiences were reflected in a recent survey of ROs in the United Kingdom, which highlighted the need for additional guidance on the safe use of AI-generated contours in clinical practice through, for example, the development of a mock dataset exemplifying typical AI failure cases [7].

### Future directions

Given the resource demanding nature of auto-contouring validation at these early stages of clinical implementation, it has been suggested that a multi-center collaborative approach to model validation may allow for a more equitable access to AI while reducing the resource burden on individual departments [7]. National Radiological bodies can lead campaigns to lobby government for such an initiative, where a centralized program is tasked with collecting and validating data across many institutions. The expected benefits include: standardization of assessment methodologies; verification of model generalizability across different institutions with more data than any single institution can collect; and a reduction of clinical resources devoted to validation. A case study example is the statewide RapidPlan rollout facilitated by the Victorian Public Sector RapidPlan Group, which formed a committee to help manage the specific data collection and validation strategies [64].

On a broader scale, the radiation oncology discipline would benefit from the creation of a high-quality benchmarking dataset—similar to those used in large language model benchmarking. The data may be partitioned according to different characteristics such as CT vendor or population demographics. Researchers would then be better positioned to design models that meet clinical needs, with the strengths and weaknesses of their models better understood in a standardized, fair and reliable manner. Importantly however, these datasets cannot be used for model training, and do not preclude internal institutional testing.

A recent investigation into geographic bias of auto-contouring models showed that no statistically significant differences in qualitative scoring were noted for a model trained on a European population and applied to an Asian population [65]. As the first investigation of auto-contouring generalizability of its kind, the results are promising but need to be extended to other anatomical sites and nationalities. Similar assessments into other population and acquisition characteristics such as BMI, age, or CT vendor are warranted to add to the body of evidence for AI generalizability.

Continued validation of APL and S-DSC, and development of more sophisticated methods of quantitative analysis can help reduce the clinical resource burden of assessing contour quality using qualitative, time-saving, and dosimetric methods. The use of quantitative metrics to correlate with time saving [25], to determine optimal metric thresholds relating to clinical suitability [44], and building models which predict qualitative scoring from quantitative metric values [66] are steps in the right direction for increasing the utility of quantitative methods.

## Conclusion

The deep learning class of algorithms is new to clinical Radiation Oncology and presents novel challenges and risks to both clinicians and patients if not adequately managed. A survey of the literature has revealed a growing set of best practices and recommendations for departments looking to implement auto-segmentation models clinically. This work synthesizes both in-house experience and the latest developments in AI standards of practice to provide a comprehensive set of selection criteria. Six vendors were retrospectively assessed in this study, revealing gaps in the market regarding desirable criteria for safe clinical use.

## Supplementary Information

Below is the link to the electronic supplementary material.

Supplementary file1 (DOCX 5698 KB)

## Data Availability

The data cannot be made publicly available upon publication because they contain sensitive personal information. The data that support the findings of this study are available upon reasonable request from the authors.
