# Supplementary material for: Guidance on selecting and evaluating AI auto-segmentation systems in clinical radiotherapy: insights from a six-vendor analysis
Source: Phys Eng Sci Med. 2025 Jan 13;48(1):301–16. doi: 10.1007/s13246-024-01513-x (PMC11997002; doi:10.1007/s13246-024-01513-x)
Supplement: Supplementary file 1 — Supplementary file1 (DOCX 5698 KB) [file 13246_2024_1513_MOESM1_ESM.docx]

**Guidance on Selecting and Evaluating AI Auto-segmentation Systems in Clinical Radiotherapy: Insights from a Six-vendor Analysis**

Branimir Rusanov^1,2,3*^, Martin A Ebert^1,2,3,4,5^, Mahsheed Sabet^1,2,3^, Pejman Rowshanfarzad^1,3^, Nathaniel Barry^1,3^, Jake Kendrick^1,3^, Zaid Alkhatib^2^, Suki Gill^2^, Joshua Dass^2^, Nicholas Bucknell^2^, Jeremy Croker^2^, Colin Tang^2^, Rohen White^2^, Sean Bydder^2^, Mandy Taylor^2^, Luke Slama^2^, Godfrey Mukwada^1,2^

*^1^School of Physics, Mathematics and Computing, The University of Western Australia, Crawley, Western Australia, Australia.*

*^2^Department of Radiation Oncology, Sir Charles Gairdner Hospital, Nedlands, Western Australia, Australia.*

*^3^Center for Advanced Technologies in Cancer Research, Perth, Western Australia, Australia.*

*^4^Australian Centre for Quantitative Imaging, The University of Western Australia, Crawley, Western Australia, Australia*

*^5^School of Medicine and Public Health, University of Wisconsin, Madison WI, USA*

*** Correspondence:**

Corresponding Author

branimir.rusanov@research.uwa.edu.au

**Supplementary Material**

**1 Quantitative Evaluation**

**1.1 Imaging Data**

A retrospective selection of 20 HN, 20 thoracic, and 19 male pelvis patients were identified for this study, which was performed under approval RGS4979 from the Sir Charles Gairdner Osbourne Park Hospital Group Human Research Ethics Committee. Patients were sampled to include institutionally relevant heterogeneity in acquisition protocols and patient characteristics. A summary of scanning protocols and patient characteristics is described in Table 1A.

All CT images were sent to Eclipse (Varian, CA) or Precision (Accuray, WI) treatment planning systems for manual contouring and planning. Contouring was initially performed by Planning Radiation Therapists (RTs) and subsequently modified and approved for clinical treatment by experienced Radiation Oncologists (ROs) (SG, JD, NB, JC, CT, RW, SB, MT). Since these were clinical contour sets, not every patient had a consistent set of manual contours available for evaluation. Table 2A shows the OARs investigated and the associated sample size for each site. Auto-contours were generated for each patient using each of the six vendor solutions.

Table 1A. Scanning protocol and patient characteristics.

| Site | Head and Neck | | Thorax | | | Male Pelvis | |
| --- | --- | --- | --- | --- | --- | --- | --- |
| CT Vendor | Toshiba Aquilion LB | | | | | | |
| kVp | 120 | | | | | | |
| mAs | Variable | | | | | | |
| N_patients_ | 20 | | 20 | | | 19 | |
| Voxel Spacing | 1.07 x 1.07 x 2.00mm | 1.37 x 1.37 x 2.00mm | 1.07 x 1.07 x 2.00mm | 1.37 x 1.37 x 2.00mm | 1.07 x 1.07 x 1.00mm | 1.07 x 1.07 x 2.00mm | |
| Imaging Heterogeneity | Standard CT (N = 20) | | Standard CT (N = 10) | Average intensity projection of 4DCT (N = 4) | CT for CyberKnife (N = 6) | Standard CT (N = 17) | Metal artefact reduction (SEMAR) (N = 2) |
| Patient Heterogeneity | Post Operative (N = 4) | | Oesophagus (N = 4); Lung (N = 16) | | | False Hip (N = 4); Prostatectomy (N = 1); Rectal Spacer Gel (N = 4) | |

Table 2A. Total number of manual contours for each structure under investigation. Symmetric organs are grouped and summed together.

| Head and Neck | |
| --- | --- |
| Structure | Number |
| Brainstem | 16 |
| Cochlea L/R | 17 |
| Eye L/R | 32 |
| Larynx | 16 |
| Lens L/R | 33 |
| Mandible | 20 |
| Oesophagus | 17 |
| Oral Cavity | 20 |
| Optic Chiasm | 6 |
| Optic Nerve L/R | 22 |
| Parotid L/R | 35 |
| Pharyngeal Constrictor | 20 |
| Spinal Cord | 15 |
| Submandibular Gland L/R | 25 |
| Thorax | |
| Carina | 12 |
| Chestwall L/R | 10 |
| Heart | 20 |
| Kidney L/R | 8 |
| Liver | 5 |
| Lung L/R | 40 |
| Oesophagus | 14 |
| Spinal Cord | 20 |
| Male Pelvis | |
| Bladder | 19 |
| Femur L/R | 31 |
| Femur L/R (Metal) | 7 |
| Prostate | 9 |
| Rectum | 18 |
| Sigmoid | 14 |

**1.2 Preprocessing**

All patient data was de-identified prior to study commencement. CT images, along with six auto-contour sets and a single manual contour Radiotherapy Structure Set (RTSS) data were obtained for binary map conversion. RTSS DICOM files contain a consecutive series of discrete points which specify the 2D polygon representation of the contour. The user may either operate directly on the 2D polygon representation (as described in [1] and accessible from <https://github.com/Auto-segmentation-in-Radiation-Oncology/Chapter-15>), or convert RT-Struct into binary voxel maps. Open-source solutions for voxelization include Plastimatch [2], RT_Utils [3], Platipy [4], DicomRTTool [5], PyDicom [6], Med-Image Tools [7], and PyRaDiSe [8].

In our investigation, we noted a difference in conversion between RT_Utils based voxelization, which is used in most python libraries, and conversions performed using Plastimatch. Figure 1A demonstrates this difference for an exaggerated scenario of a very small segment of several millimeters across. As can be seen, Plastimatch more faithfully conforms to the polygon while RT_Utils based conversions miss a large portion of the contour (left in Figure 1A), or overshoot the ground truth contour (right in Figure 1A). For this reason, we used Plastimatch when converting from RT-struct to voxel representations.

Automatically generated contours had consistent naming conventions, whereas manual contours contained discrepancies such as spelling errors, abbreviations, and varying spacing. To automate the selection of correct contours from all datasets, string similarity metrics (cosine distance and normalized Lavenshtein) were employed to parse through each patient contour directory and pull individual contour NIFTI files with the highest score with respect to a template naming convention. Logs for each patient were generated and inspected to ensure correct contour capture. All contours were then loaded onto memory and converted into NumPy arrays for quantitative evaluation.


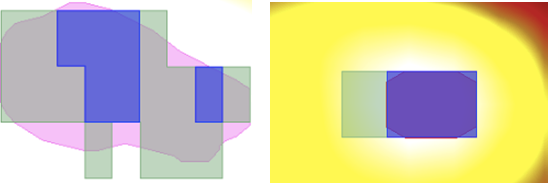


Figure 1A. LEFT: Blue = RT_Utils; Green = Plastimatch; Red = Truth; RIGHT: Green = RT_Utils; Blue = Plastimatch; Red = Truth

**1.3 Metric Computation**

A total of five quantitative metrics were computed for our dataset: Added Path Length (APL), Surface Dice (S-DSC), Hausdorff Distance-95^th^ Percentile (HD95), Average Surface Distance (ASD), and Dice-Soerensen Coefficient (DSC). Figures 2A – 6A summarize box and whisker plots for head and neck (HN) organs at risk (OAR), Figures 7A – 11A for pelvic OAR, and Figures 12A – 16A for the thoracic OAR.

Due to potential differences in metric implementation, the source of the five metrics used in this study are briefly summarized: DSC, S-DSC, HD95, and ASD were employed using the Google DeepMind implementation [9]. APL was implemented using code from the PlatiPy [4] library. Metric results were saved into Pandas DataFrames and exported into Excel for analysis. For structures with ambiguously defined superior and inferior extents (spinal cord, oesophagus, rectum, sigmoid), auto-contoured datasets were truncated to match manual contour extents to avoid penalizing models that contoured beyond the typical protocol at our institution.

**1.4 Scoring Heuristic**

A scoring heuristic was devised to simplify the presentation of quantitative results, which may otherwise be difficult to absorb at once. The heuristic considers the mean of the best performing model, which is assigned a 1, and worst performing model, which is assigned a 0, for a given structure and metric. Both S-DSC and APL are assessed, such that the best possible score for a given structure is 2 (*i.e.* a model had the best mean in terms of S-DSC and APL). All intermediate models are assigned a linear score based on their position relative to the best and worst results. To prevent penalizing intermediate scoring models with results similar to the best performing model, the standard error (+/-) of intermediate models was computed. If the mean of the best performing model fell within the mean +/- standard error of an intermediate model, then the intermediate model was assigned a score of 1. Lastly, if a vendor did not offer a particular organ, it was denoted as N/A and scored as zero. The scoring heuristic results for HN, thoracic and pelvic regions are summarized in Tables 3A – 5A.

Table 3A. Scoring heuristic results for the HN region.

|  | Vendor | | | | | |
| --- | --- | --- | --- | --- | --- | --- |
| Structure | A | B | C | D | E | F |
| Brainstem | 1.57 | 0.00 | 0.90 | 2.00 | 0.77 | 1.46 |
| Chiasm | 2.00 | 1.75 | 1.13 | 0.60 | 0.00 | 2.00 |
| Cochlea_L/R | 0.00 | 0.30 | N/A | 0.67 | 0.38 | 2.00 |
| Eye_L/R | 1.55 | 1.00 | 0.47 | 0.54 | 2.00 | 2.00 |
| Larynx | 2.00 | 1.80 | 0.61 | N/A | 0.59 | 0.00 |
| Lens_L/R | 1.00 | 2.00 | 1.23 | 2.00 | 2.00 | 2.00 |
| Mandible | 2.00 | 0.45 | 0.38 | 1.76 | 2.00 | 2.00 |
| Oesophagus | 1.75 | 1.61 | 1.70 | N/A | 0.00 | 2.00 |
| Optic Nerve_L/R | 1.14 | 1.24 | 0.65 | 0.00 | 0.85 | 2.00 |
| Oral Cavity | 1.26 | 0.32 | 1.00 | 2.00 | 2.00 | 1.06 |
| Parotid_L/R | 2.00 | 0.00 | N/A | 0.60 | 1.67 | 1.59 |
| Pharyngeal Constrictor | 2.00 | 1.00 | N/A | N/A | 1.00 | N/A |
| Spinal Cord | 2.00 | 0.37 | 0.00 | 2.00 | 1.75 | 2.00 |
| SubmanG_L/R | 2.00 | 1.06 | N/A | 1.49 | 2.00 | 0.00 |
| TOTAL | 22.3 | 12.9 | 8.07 | 13.7 | 17.0 | 20.1 |

Table 4A. Scoring heuristic results for the thoracic region.

|  | Vendor | | | | | |
| --- | --- | --- | --- | --- | --- | --- |
| Structure | A | B | C | D | E | F |
| Carina | n/a | 2.00 | N/A | 0.00 | N/A | 0.49 |
| Chestwall_L/R | 2.00 | 0.12 | N/A | N/A | 0.69 | 0.02 |
| Heart | 2.00 | 2.00 | 1.30 | 0.00 | 0.15 | 2.00 |
| Kidney_L/R | 2.00 | N/A | 0.00 | 1.27 | 2.00 | 1.69 |
| Liver | 2.00 | 0.21 | 0.00 | 0.16 | 2.00 | 1.34 |
| Lung_L/R | 1.22 | 1.77 | 0.00 | 2.00 | 1.38 | 1.22 |
| Oesophagus | 1.00 | 0.36 | 2.00 | 2.00 | 2.00 | 2.00 |
| Spinal Cord | 2.00 | 1.44 | 0.00 | 0.84 | 1.52 | 2.00 |
| TOTAL | 12.2 | 7.90 | 3.30 | 6.27 | 9.74 | 10.8 |

Table 5A. Scoring heuristic results for the pelvic region.

|  | Vendor | | | | | |
| --- | --- | --- | --- | --- | --- | --- |
| Structure | A | B | C | D | E | F |
| Prostate | 2.00 | 0.20 | 0.00 | 2.00 | 2.00 | 1.44 |
| Bladder | 1.86 | 1.05 | 0.00 | 1.77 | 2.00 | 2.00 |
| Femur L/R | 1.08 | 1.85 | 0.00 | 2.00 | 2.00 | 2.00 |
| Femur L/R (Metal) | 0.72 | 1.45 | 0.00 | 2.00 | N/A | 1.49 |
| Rectum | 2.00 | 0.14 | 0.32 | 1.68 | 2.00 | 1.00 |
| Sigmoid | N/A | N/A | N/A | 2.00 | 0.00 | 2.00 |
| TOTAL | 7.66 | 4.69 | 0.320 | 11.5 | 8.00 | 9.93 |


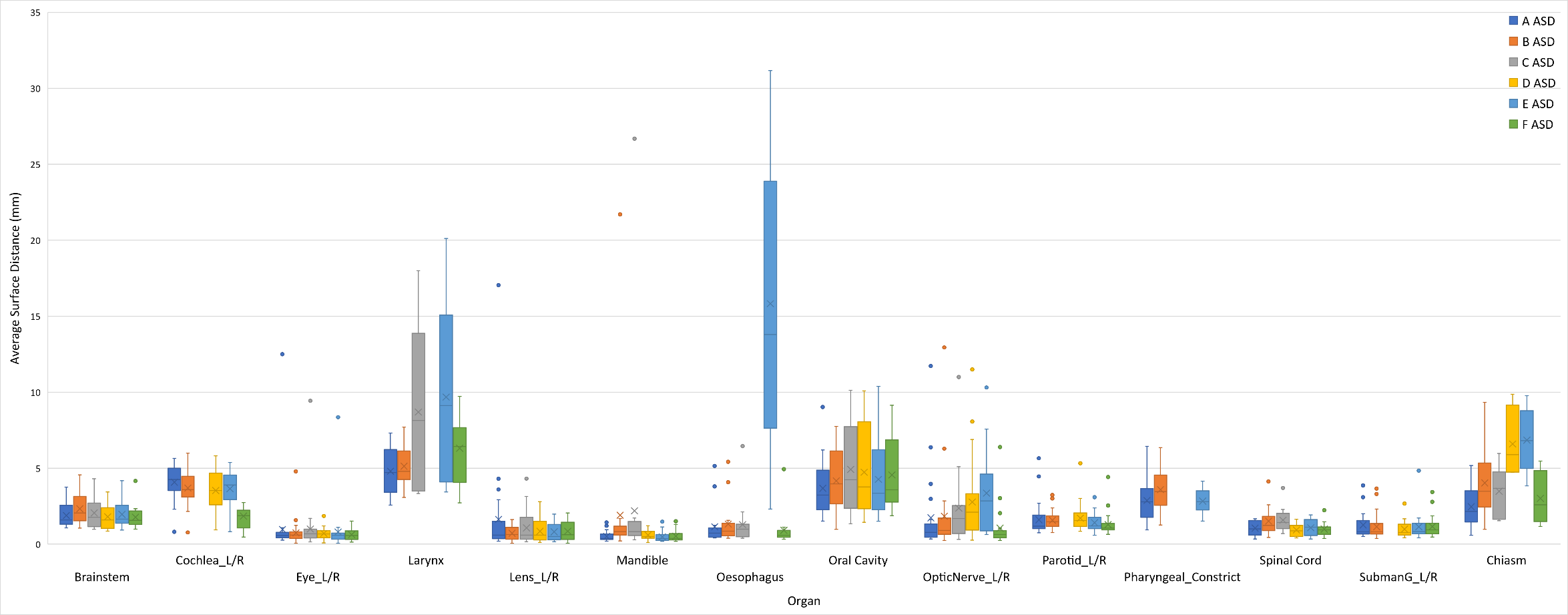


Figure 2A. Average Surface Distance (mm) box plot for six vendors compared against manual contours for head and neck organs.


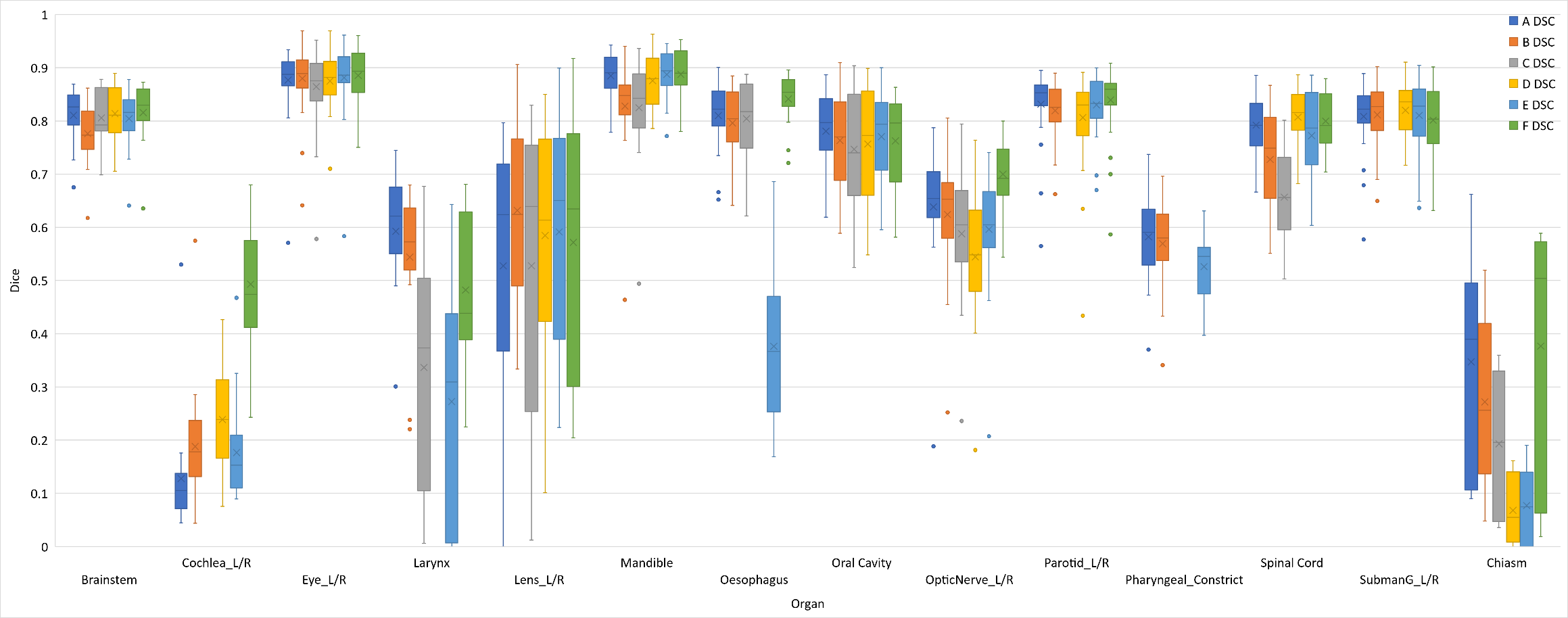


Figure 3A. Dice coefficient for box plot for six vendors compared against manual contours for head and neck organs.


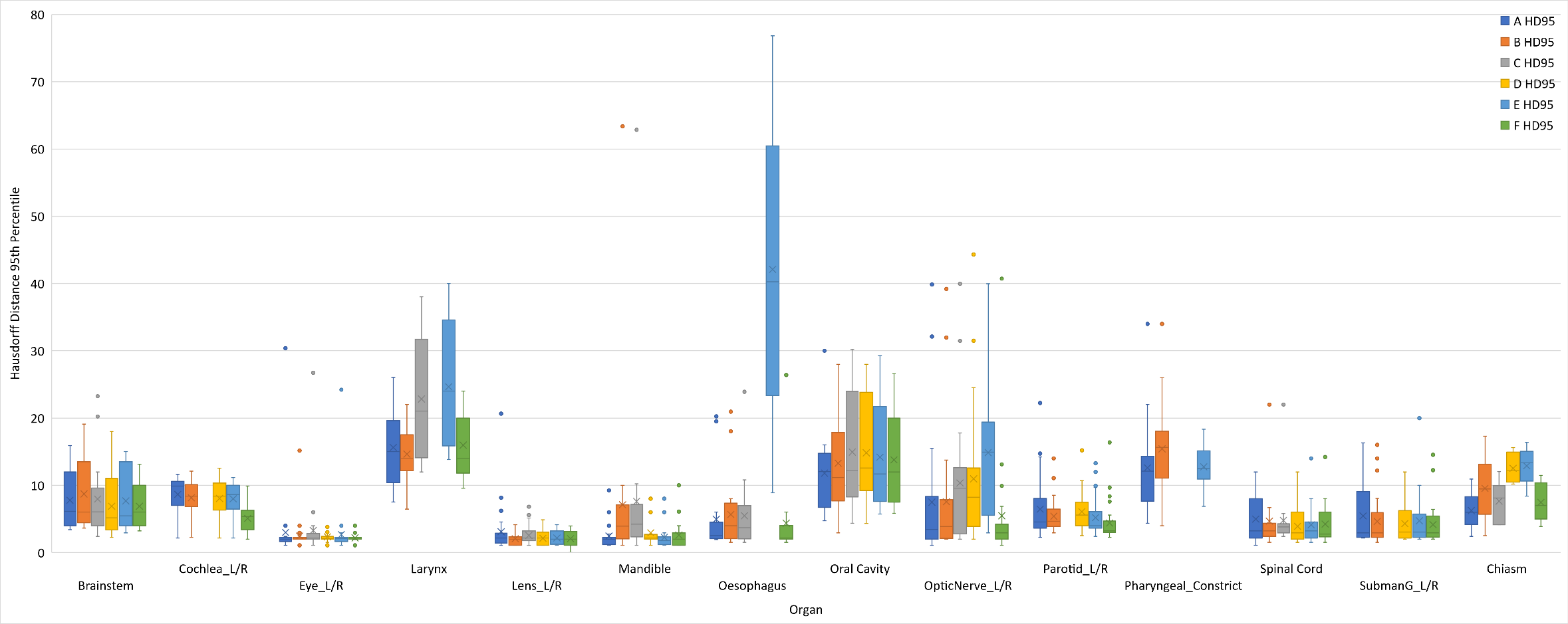


Figure 4A. Hausdorff Distance 95^th^ Percentile (mm) box plot for six vendors compared against manual contours for head and neck organs.


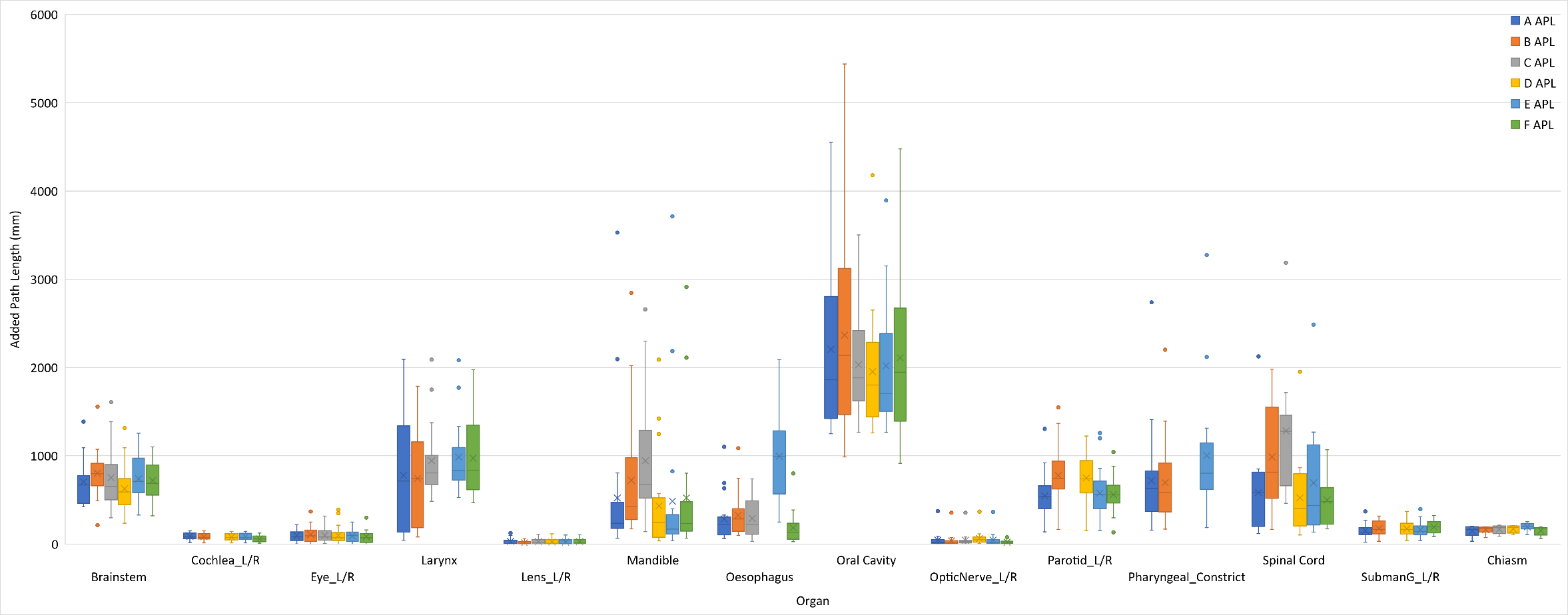


Figure 5A. Added path length (mm) box plot for six vendors compared against manual contours for head and neck organs.


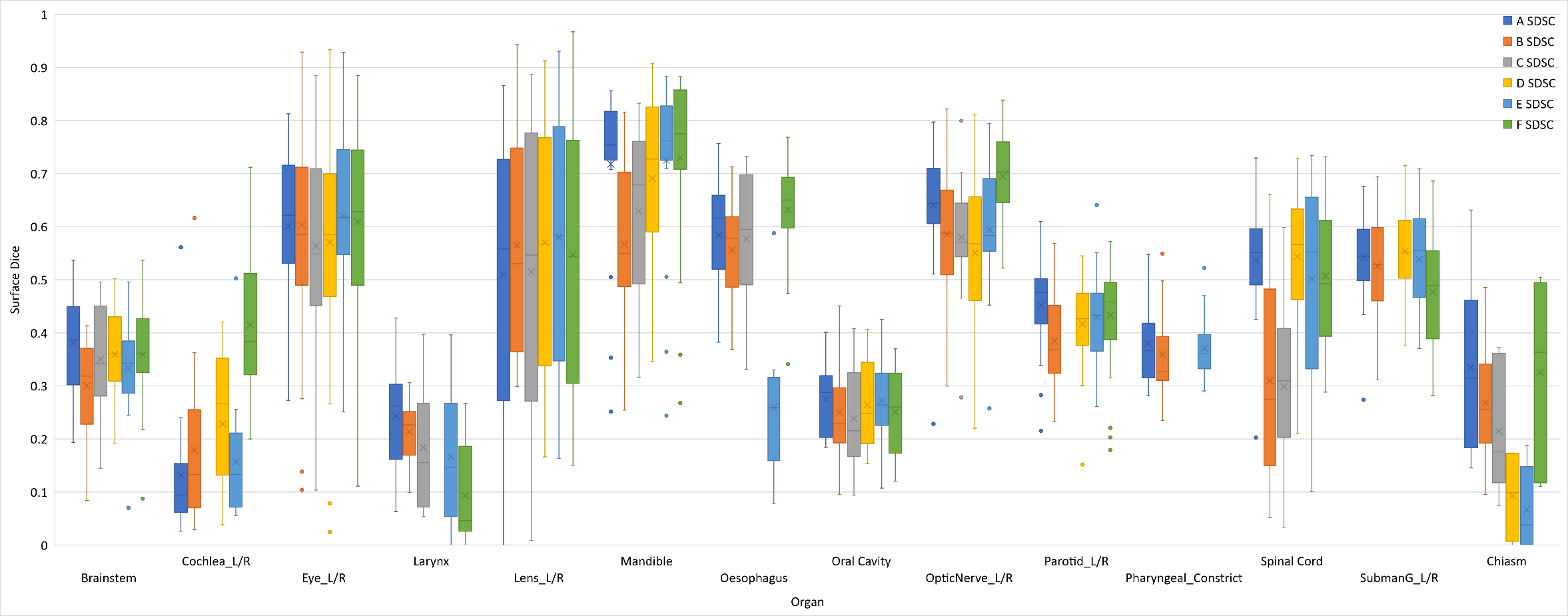


Figure 6A. Surface DICE box plot for six vendors compared against manual contours for head and neck organs.


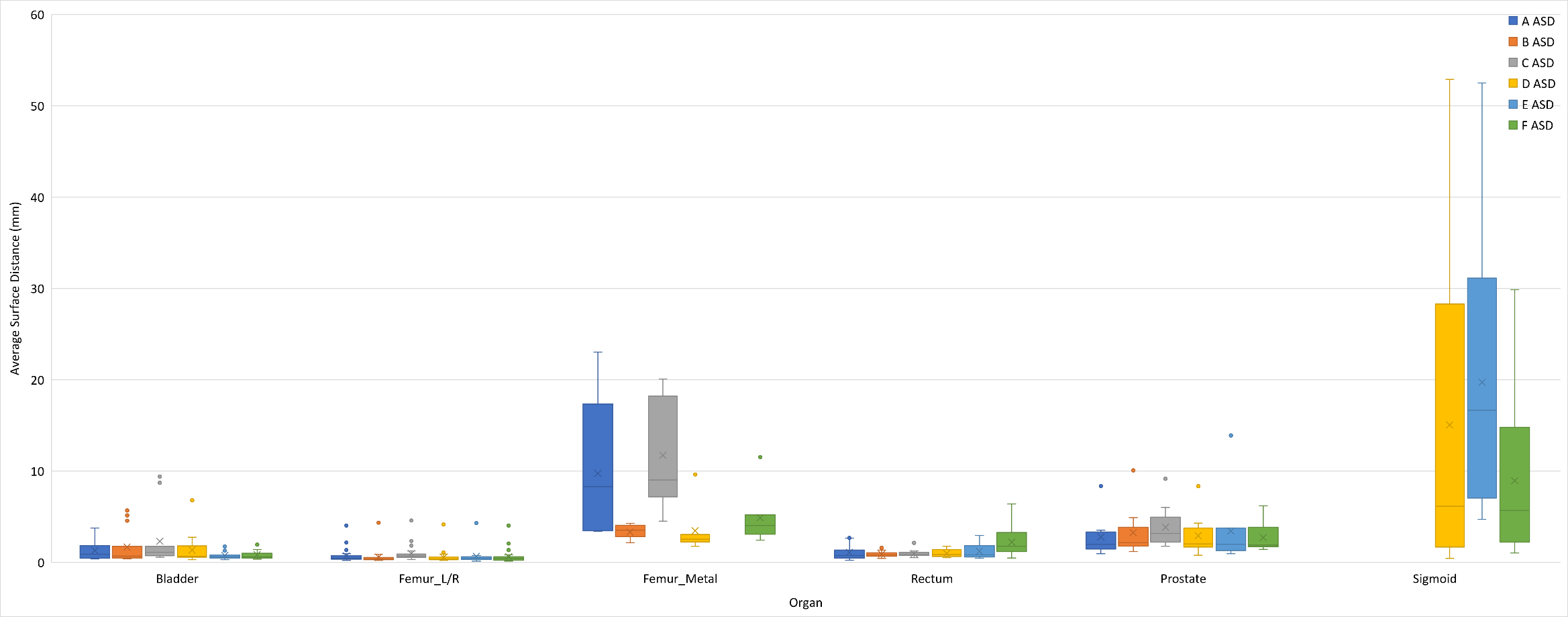


Figure 7A. Average Surface Distance (mm) box plot for six vendors compared against manual contours for pelvic organs.


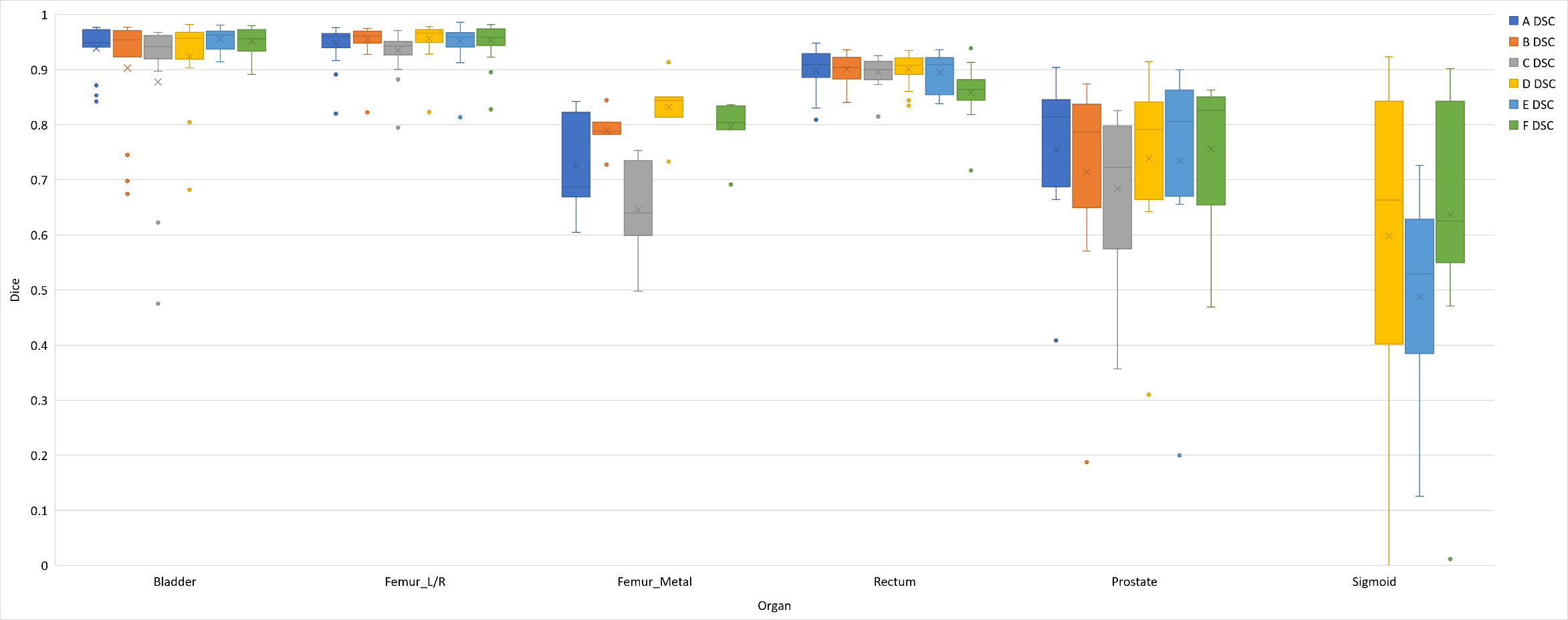


Figure 8A. Dice coefficient box plot for six vendors compared against manual contours for pelvic organs.


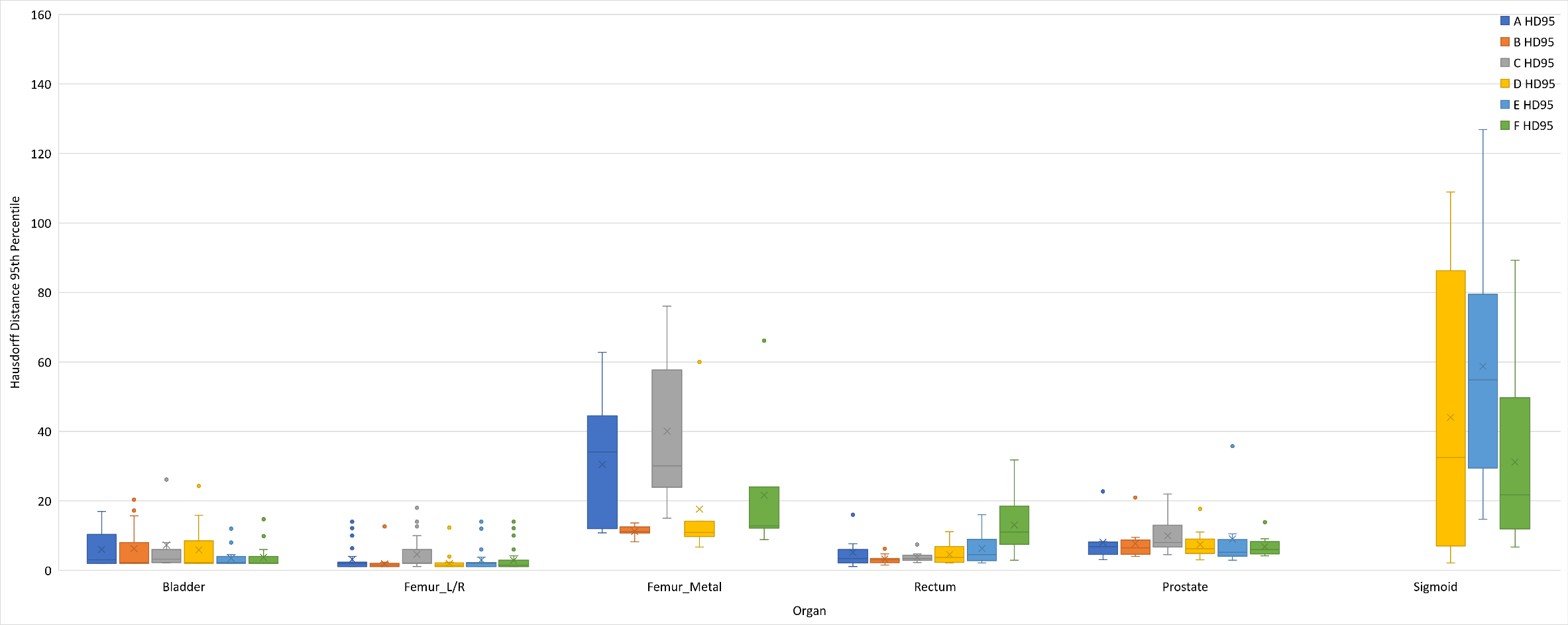


Figure 9A. Hausdorff distance 95^th^ percentile (mm) box plot for six vendors compared against manual contours for pelvic organs.


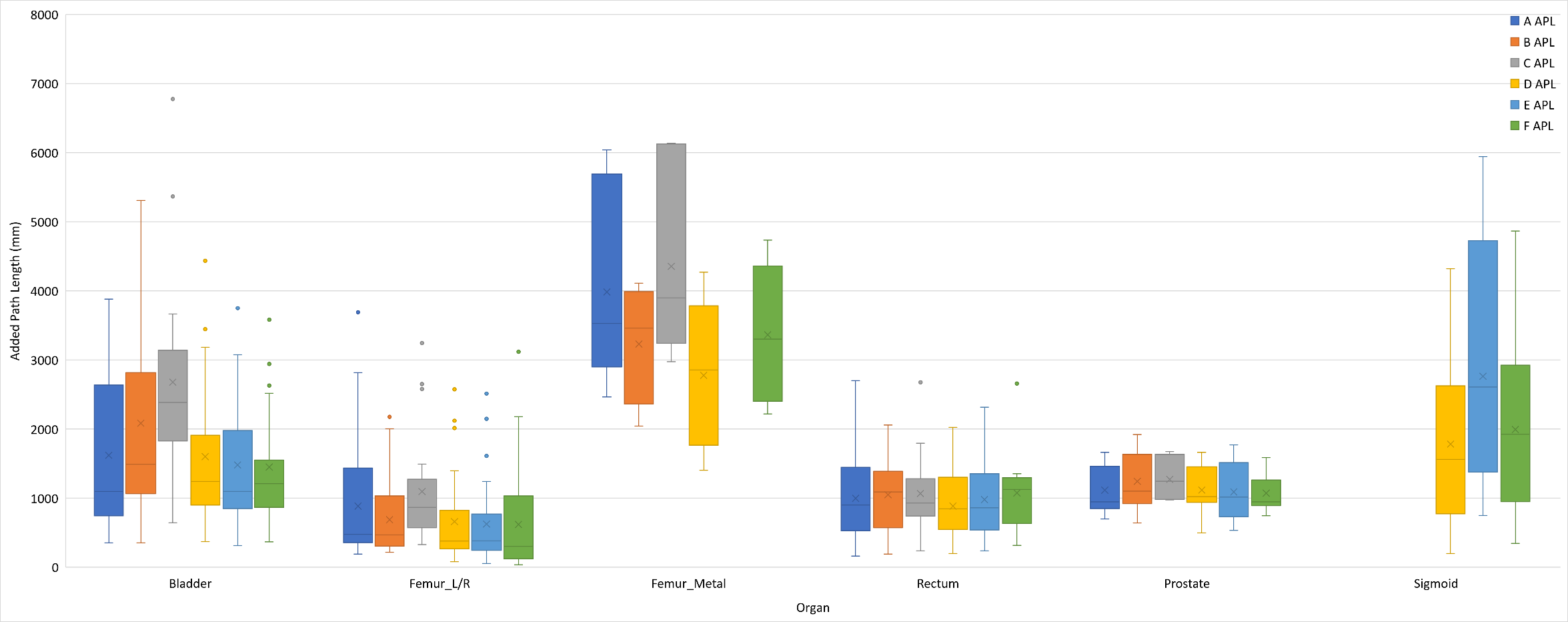


Figure 10A. Added path length (mm) box plot for six vendors compared against manual contours for pelvic organs.


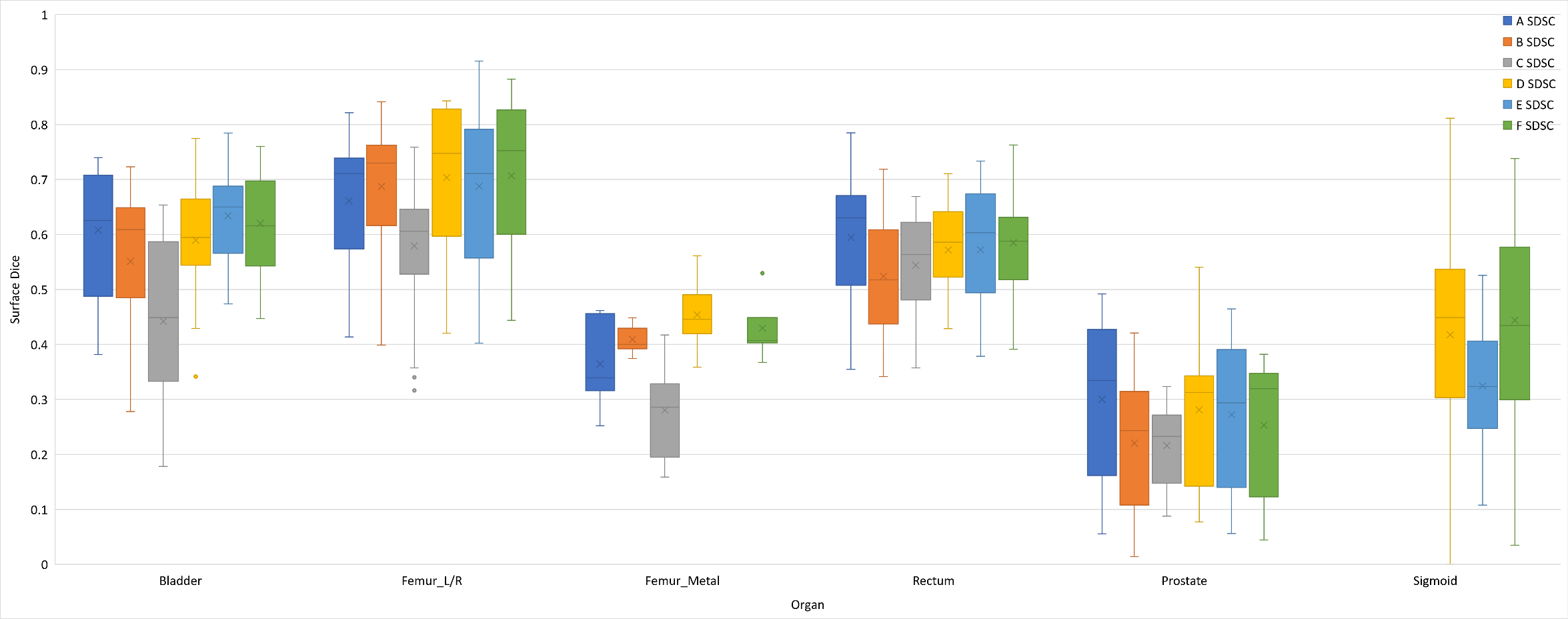


Figure 11A. Surface DICE box plot for six vendors compared against manual contours for pelvic organs.

.


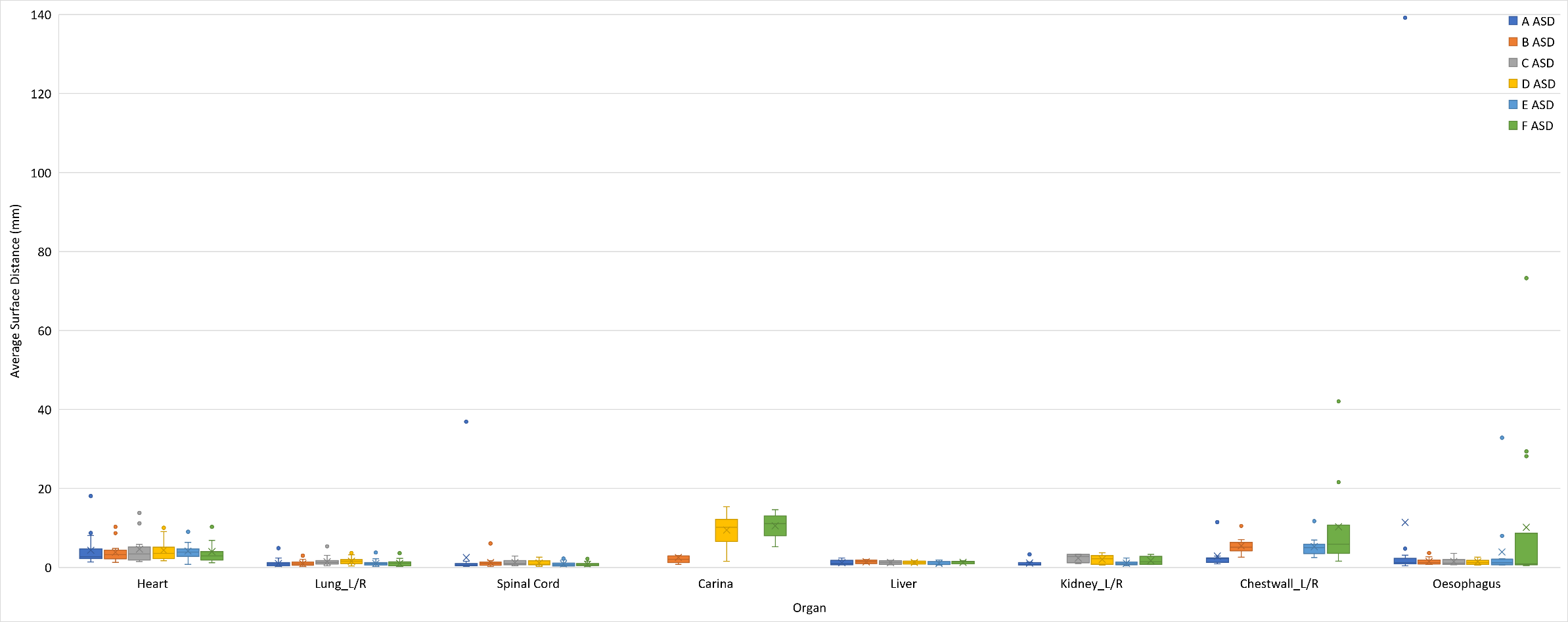


Figure 12A. Average Surface Distance (mm) box plot for six vendors compared against manual contours for thoracic organs.


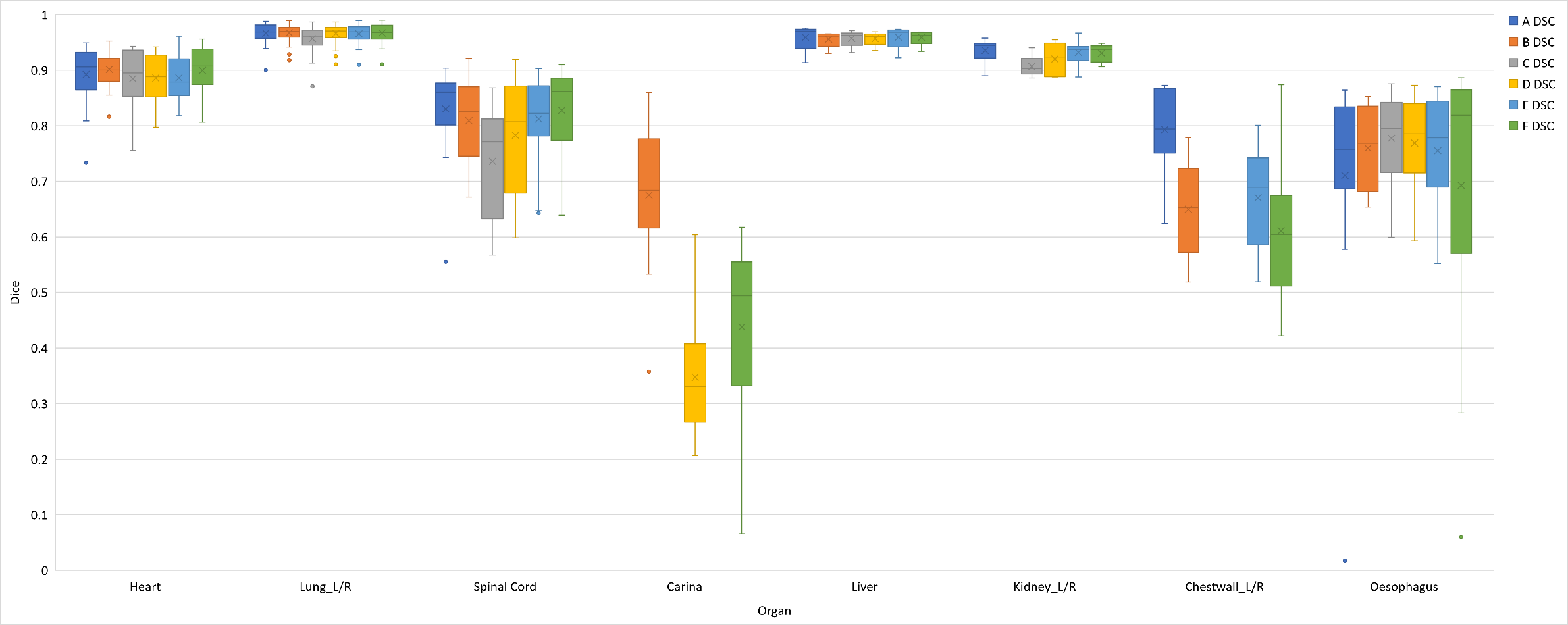


Figure 13A. Dice coefficient box plot for six vendors compared against manual contours for thoracic organs.


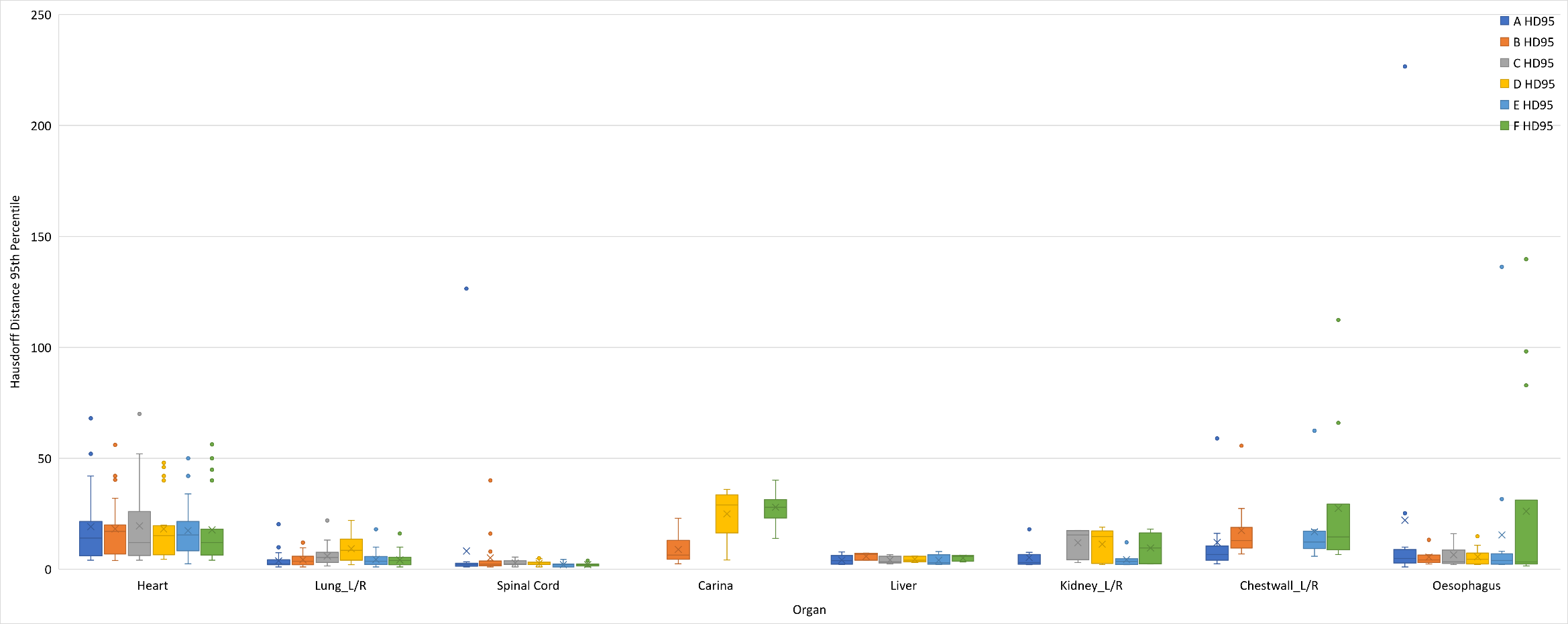


Figure 14A. Hausdorff Distance 95^th^ Percentile (mm) box plot for six vendors compared against manual contours for thoracic organs.


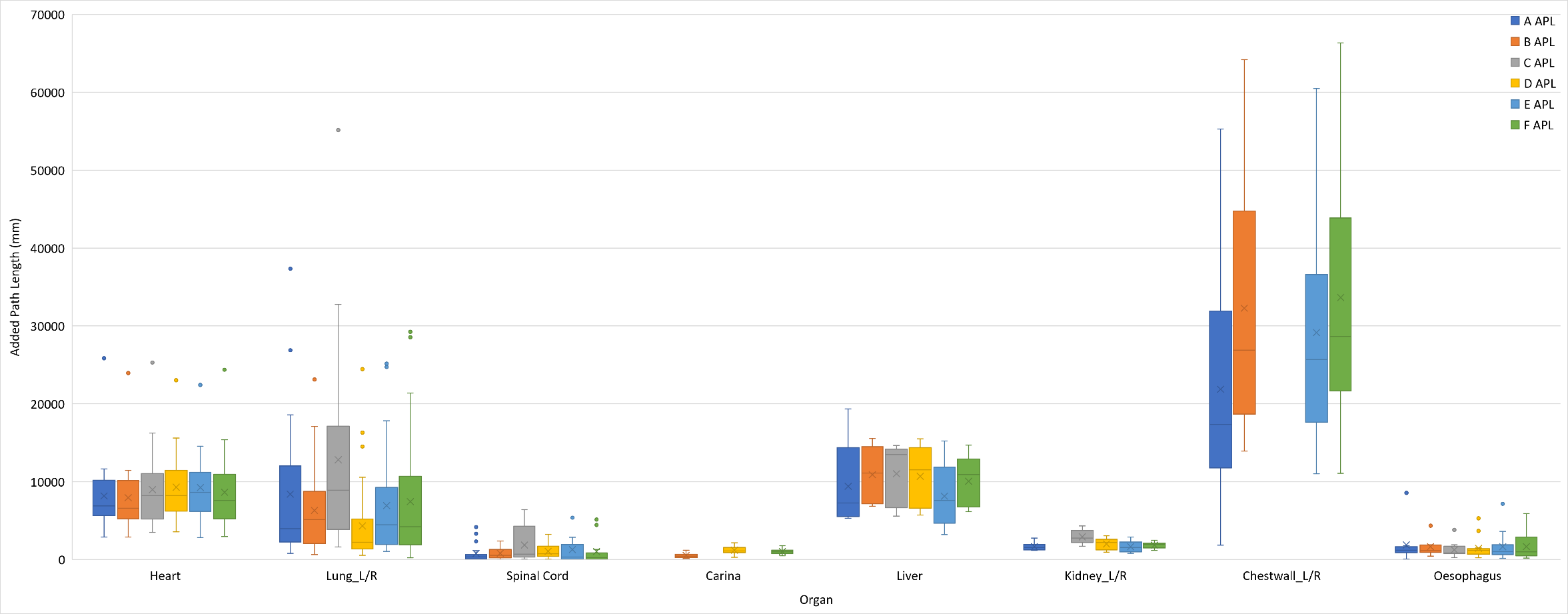


Figure 15A. Added path length (mm) box plot for six vendors compared against manual contours for thoracic organs.


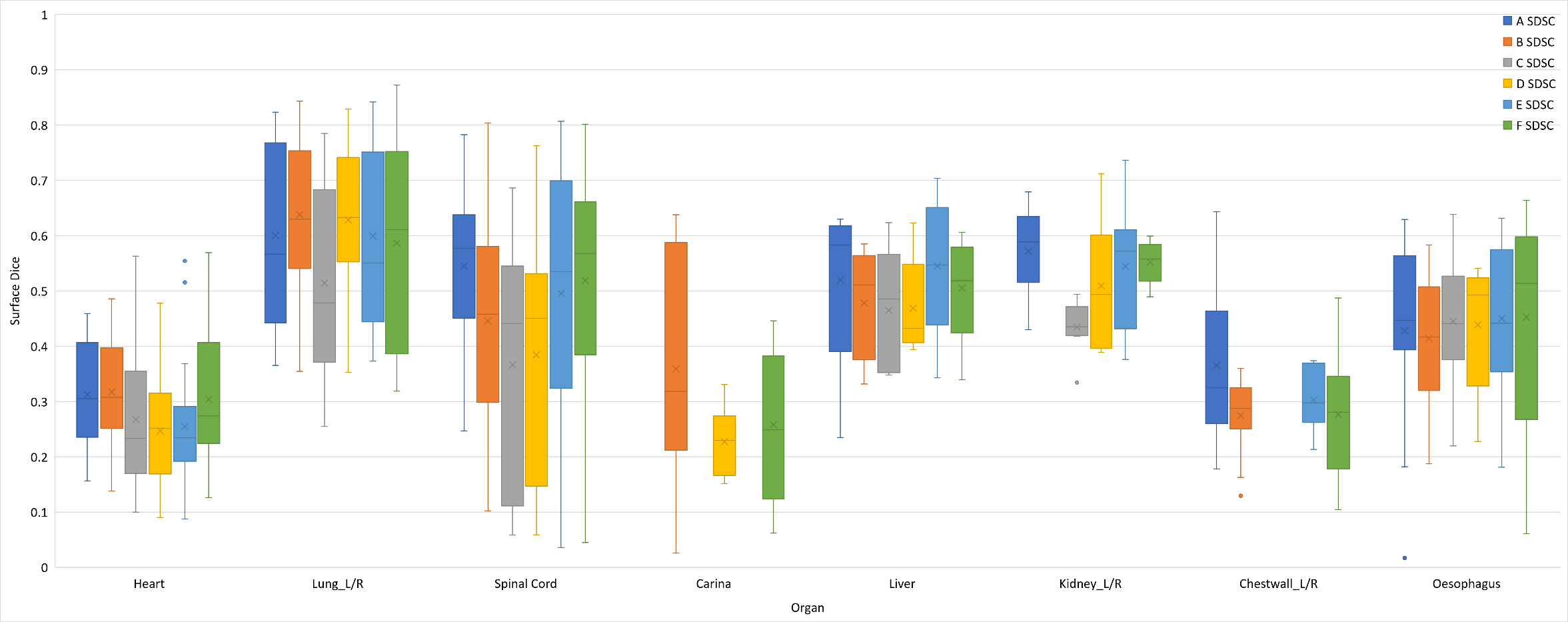


Figure 16A. Surface DICE box plot for six vendors compared against manual contours for thoracic organs.

**1.5 Quantitative Comparison with Doolan et al.**

Recently Doolan et al. [10] published quantitative results for five commercial AI auto-contouring models offered by Mirada, MVision, Radformation, Raystation, and Therapanacea for model versions as of April 2022. Table 6A summarizes the range of median Dice coefficients for 23 common structures between their study and ours. Only three structures had non-overlapping ranges between both evaluations: the prostate (median range 0.854 – 0.905 [10] vs 0.722 – 0.826), the heart (median range 0.919 – 0.950 [10] vs 0.879 – 0.907), and left/right femoral heads (0.883 – 0.913 [10] vs 0.943 – 0.966). Two factors were identified for prostate disagreement: (1) four patients had rectal spacer gel which reduced auto-contouring accuracy of the prostate structure, (2) three patients had greater superior and inferior extents of the prostate due to delineation on MRI imaging. Heart disagreement was due to the greater superior extent of heart segmentation at our institution compared to auto-contouring models. Femoral head disagreement could be due to differences in the definition of the inferior femoral head extent. Nevertheless, disagreements were small and overall corroboration of results between both works provided reassurance of the correctness of assessment methods for each approach.

Table 6A. The range of median DSC values for the five vendors assessed in Doolan et al. compared to the current study.

| Structure | Doolan et al. | Rusanov et al. |
| --- | --- | --- |
| Head and Neck | | |
| Brainstem | 0.786 – 0.889 | 0.773 – 0.830 |
| Eye_L/R | 0.891 – 0.925 | 0.875 – 0.893 |
| Lens_L/R | 0.561 – 0.830 | 0.676 – 0.792 |
| Mandible | 0.856 – 0.926 | 0.842 – 0.894 |
| Parotid_L/R | 0.818 – 0.880 | 0.826 – 0.859 |
| Spinal Cord | 0.687 – 0.834 | 0.656 – 0.816 |
| SubmanG_L/R | 0.750 – 0.893 | 0.804 – 0.836 |
| Thorax | | |
| Heart | 0.919 – 0.950 | 0.879 – 0.907 |
| Liver | 0.960 – 0.971 | 0.961 – 0.970 |
| Lung_L/R | 0.946 – 0.971 | 0.961 – 0.971 |
| Oesophagus | 0.539 – 0.846 | 0.758 – 0.819 |
| Spinal Cord | 0.790 – 0.842 | 0.771 – 0.861 |
| Pelvis | | |
| Bladder | 0.947 – 0.973 | 0.942 – 0.963 |
| Femur_L/R | 0.883 – 0.913 | 0.943 – 0.966 |
| Prostate | 0.854 – 0.905 | 0.722 – 0.826 |
| Rectum | 0.826 – 0.906 | 0.864 – 0.909 |
| Sigmoid | 0.521 – 0.770 | 0.529 – 0.664 |

**2 Qualitative Evaluation**

Auto-contours were blindly scored by three experienced radiation oncologists (ROs), (NB: < 5 years’ experience, CT: > 10 years’ experience, JC 5 – 9 years’ experience). Table 7A shows the distribution of scoring performed for each site and RO. Due to the large number of structures to assess, RO_2_ and RO_3_ only assessed four out of six vendors. RO_1_ and RO_2_ both assessed three unique patients each, while RO_3_ assessed only one unique patient. For each case, raters were blinded to the source of the auto-contour vendor. A 5-point scoring criteria was defined as:

1. Structure is completely unusable OR was not contoured (but should have been) – I would erase it and recreate it from scratch.
2. Major adjustments required – expect little to no time will be saved compared to manual drawn contours.
3. Moderate adjustments required – expect some time will be saved.
4. Minor adjustments required – expect significant time saved.
5. Structure is clinically acceptable.

After scoring all patients, the ROs were asked to select their preferred auto-contour source.

**2.1 Qualitative Analysis**

The results of the blinded qualitative study are summarized in Table 7A. Overall, the HN region demonstrated the highest accuracy among vendors, with 4/6 achieving clinically acceptable contours (score ≥ 4) for over 80% of the structures analyzed. Raters were less satisfied with pelvic auto-contours, where only half of vendors achieved acceptability greater than 80%. The thoracic region was only assessed for a subset of vendors, with 2/4 scoring above 80%. Overall, none of the assessed vendors consistently outperformed the others based on RO scoring, however, vendors A, B, D, and F scored above 80% for at least 2/3 sites. In terms of overall RO preference, vendors A and F were equally preferred for the HN site, while vendor A was preferred for the thorax site. No vendor was preferred over any other for the pelvic region.

Table 8A summarizes the quantitative and qualitative results used to assess the performance of the six vendors. A color map shows the agreement between qualitative and quantitative results, while the Pearson Correlation Coefficient is included to demonstrate the numerical agreement. Disagreements are expected as quantitative results were formed from a much larger cohort of patients and OARs, while qualitative assessments represented a limited sample size. Nevertheless, a very high and high agreement is observed for the HN and pelvic sites, respectively, while the thorax showed a moderate-weak correlation likely due to the smaller rater and sample size. Importantly, “best-performing” models were accurately captured by both quantitative and qualitative methods.

Table 7A. RO scoring distribution and qualitative scores equal to or greater than 4. RO_1_ and RO_2_ each assessed three unique patients each. RO_3_ assessed one unique patient.

| Site | HN | Thoracic | Pelvic |
| --- | --- | --- | --- |
| RO_1_ | A, B, C, D, E, F |  | A, B, C, D, E, F |
| RO_2_ | A, B, D, F | A, B, D, F |  |
| RO_3_ |  |  | A, B, D, F |
| % Score ≥ 4 | A: 91%, B: 83%, C: 63%, D: 61%, E: 92%, F: 83% | A: 100%, B: 80%, D: 60%, F: 53% | A: 65%, B: 65%, C: 44%, D: 85%, E: 84%, F: 88% |
| RO Preferred | A/F | A | No preference. |

Table 8A. Heatmap of data correlation between quantitative and qualitative results for HN, thoracic and pelvic sites, along with the associated Pearson Correlation Coefficient.

|  | HN | | Thorax | | Pelvis | |
| --- | --- | --- | --- | --- | --- | --- |
| Vendor | Qualitative | Quantitative | Qualitative | Quantitative | Qualitative | Quantitative |
| A | 0.910 | 22.26 | 1.00 | 12.2 | 0.650 | 7.66 |
| B | 0.830 | 12.91 | 0.800 | 7.90 | 0.650 | 4.69 |
| C | 0.630 | 8.07 |  |  | 0.440 | 0.320 |
| D | 0.610 | 13.66 | 0.600 | 6.27 | 0.850 | 11.5 |
| E | 0.920 | 17.02 |  |  | 0.840 | 8.00 |
| F | 0.830 | 20.11 | 0.530 | 10.8 | 0.880 | 9.93 |
| Correlation | 0.741 | | 0.462 | | 0.927 | |

**References**

1. Yang, J., G.C. Sharp, and M.J. Gooding, *Auto-Segmentation for Radiation Oncology : State of the Art*. Series in Medical Physics and Biomedical Engineering Series. 2021, Milton: Taylor & Francis Group.

2. Sharp, G.C., et al. *Plastimatch: an open source software suite for radiotherapy image processing*.

3. Shrestha, A. *RT-Utils: a minimal Python library to facilitate the creation and manipulation of DICOM RTStructs*. 2023; Available from: <https://github.com/qurit/rt-utils>.

4. Chlap, P. and R.N. Finnegan, *PlatiPy: Processing Library and Analysis Toolkit for Medical Imaging in Python.* Journal of Open Source Software, 2023. **8**(86): p. 5374.

5. Anderson, B.M., K.A. Wahid, and K.K. Brock, *Simple python module for conversions between DICOM images and radiation therapy structures, masks, and prediction arrays.* Practical radiation oncology, 2021. **11**(3): p. 226-229.

6. Mason, D., *SU‐E‐T‐33: pydicom: an open source DICOM library.* Medical Physics, 2011. **38**(6Part10): p. 3493-3493.

7. Kim, S., et al., *Med-ImageTools: An open-source Python package for robust data processing pipelines and curating medical imaging data.* F1000Research, 2023. **12**(118): p. 118.

8. Rüfenacht, E., et al., *PyRaDiSe: A Python package for DICOM-RT-based auto-segmentation pipeline construction and DICOM-RT data conversion.* Computer methods and programs in biomedicine, 2023. **231**: p. 107374.

9. DeepMind. *DeepMind Surface Distance Metrics*. [cited 2023; Available from: <https://github.com/deepmind/surface-distance>.

10. Doolan, P.J., et al., *A clinical evaluation of the performance of five commercial artificial intelligence contouring systems for radiotherapy.* Frontiers in Oncology, 2023. **13**: p. 1213068.
